# The benefits of multi-^2^D LC × LC compared to LC × LC for the analysis of European herbal remedies

**DOI:** 10.1007/s00216-025-06278-0

**Published:** 2025-12-19

**Authors:** K. Wetzel, P. Nhan, T. Tishakova, T. Niedenthal, M. Häßler, J. F Ayala-Cabrera, L. Montero, O. J. Schmitz

**Affiliations:** 1 Applied Analytical Chemistry, University of Duisburg-Essen, Universitaetsstr. 5, 45141 Essen, Germany; 2 Forschergruppe Klostermedizin GmbH, Annastr. 26a, 97072 Würzburg, Germany; 3 Department of Analytical Chemistry, University of the Basque Country (UPV/EHU), Sarriena Auzoa, 48940 Leioa, Spain; 4 Research Centre for Experimental Marine Biology and Biotechnology, University of the Basque Country (PiE-UPV/EHU), Areatza Hiribidea 47, 48620 Plentzia, Spain; 5 Foodomics Laboratory, Institute of Food Science Research – CIAL (CSIC-UAM), Calle Nicolás Cabrera 9, 28049 Madrid, Spain

**Keywords:** Column screening, High-resolution mass spectrometry, Medicinal plants, Multi-^2^D LC × LC, Phenolic compounds

## Abstract

Due to the demand in alternative medicine to treat various diseases, medicinal plants as ancient remedies gained more recent interest for the discovery and isolation of active compounds (Int J Environ Res Public Health. 2020;17(10):3376, J Ethnopharmacol. 2017;199:161-167). As these plant matrices contain an enormous range of metabolites, the challenge lies in the selection and application of separation techniques capable of resolving such complexity. For this reason, comprehensive two-dimensional liquid chromatography (LC × LC), due to the increased peak capacity compared to one-dimensional LC, was firstly selected for the chemical characterization of five European medicinal plants (*Angelica archangelica*, *Angelica sylvestris*, *Agrimonia eupatoria*, *Sambucus ebulus*, and *Sambucus nigra*). For the method development, complimentary columns were chosen, optimized, and compared regarding peak capacity, orthogonality, and peak distribution. Moreover, a platform with even higher separation power was chosen using a PFP column in the first dimension and two complimentary columns in the second dimension, ZIC-HILIC from 0 to 20 min and polar C18 from 20 min until 60 min at the end, called multi-^2^D LC × LC. The optimized method resulted in a gain of 91% for the peak capacity, 8.2% for orthogonality, and 30% better peak distribution compared to the LC × LC methods. The hyphenation to high-resolution mass spectrometry enabled the chemical characterization of phenolic compounds and their distribution among the plants as well as potential marker substances for authentication purposes, providing the first comparative study of these five European herbal remedies.

**Graphical abstract:**

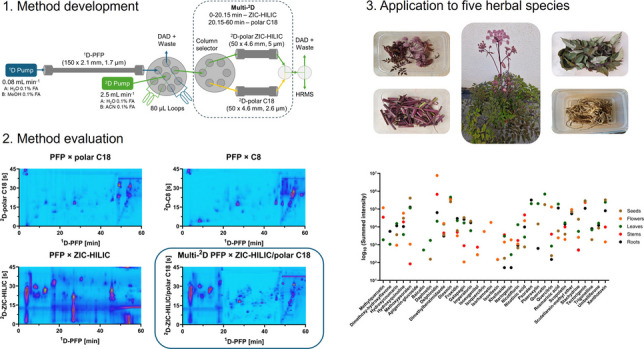

**Supplementary Information:**

The online version contains supplementary material available at 10.1007/s00216-025-06278-0.

## Introduction

Comprehensive two-dimensional liquid chromatography (LC × LC) is [1], besides GC × GC, the gold standard for the chemical analysis of complex samples as it enables increased peak capacity, resolution, and selectivity compared to one-dimensional LC [2]. Recent trends and the huge application field of this technique have been summarized by various reviews [2–6]. Even though one of the main factors is the choice of complementary separation mechanisms, the use of the reversed phase (RP) in both dimensions (RP × RP) offers good orthogonality and it is frequently used due to the broad applicability of these combinations [2, 7, 8]. The desired orthogonality in RP × RP can be achieved by applying different organic mobile phases or pH in first (^1^D) and second dimension (^2^D) or by applying parallel [9, 10] or shifting gradients in the ^2^D [7, 11]. For better selectivity, other RP phases which promote other type of interactions such as pentafluoro phenyl (PFP) proved to be a powerful combination with a C18 phase for the analysis of industrial hemp strains [12]. On the other hand, for an increased orthogonality, hydrophilic interaction chromatography (HILIC) has been widely combined with RP [13, 14] but this combination often comprises mobile phase incompatibilities that lead to analyte breakthrough [15]. Several modulation strategies have been developed to solve mobile phase mismatches such as at-column dilution [16, 17], active solvent modulation [18, 19], or the use of trapping columns [20]. As a reasonable alternative to HILIC modes, a reversed gradient using low concentrations of acetonitrile has been used for over a decade, named reversed HILIC (revHILIC) or per aqueous liquid chromatography (PALC) [21, 22]. Furthermore, a new approach consisted of installing two complementary columns in the ^2^D was introduced by Montero et al. [20] to increase, even more, the separation power of LC × LC. This approach is called multi-^2^D LC × LC and allows tuning the separation of the compounds in the ^2^D by different stationary phases, enabling higher orthogonality and peak capacity.

To evaluate orthogonality and peak capacity of LC × LC measurements as important criteria, several metrics have been invented and discussed in the last decades [23–29]. Among these metrics, the orthogonality can be more authentically described if determined by the bin-counting method where the 2D-space is divided into bins and the number of bins that are occupied is counted. Since this approach considers free space between analytes, this has proven to be more realistic than others [25]. Additionally, it is recommended to calculate the peak distribution over the 2D-space as the orthogonality determines the percentage of occupied space but not the amount of peaks that cluster within the occupied spaces.

In this context, the first comparative study of the chemical profiles of five European medicinal plants, namely *Agrimonia eupatoria*, *Angelica archangelica*, *Angelica sylvestris*, *Sambucus ebulus*, and *Sambucus nigra*, known for their hepatoprotectivity [30–34], was aimed by hyphenating LC × LC to high-resolution mass spectrometry. The chemical profiles of these herbal remedies were previously analyzed via 1D-LC [35–39] but remained challenging due to sample complexity and the concentration and polarity range of compounds. Recently, it has been demonstrated that plant extracts obtained under microwave-assisted extraction (MAE) with potential antioxidant activity presented a complex chemical composition impossible to be elucidated using conventional one-dimensional LC, and that benefited from an improved separation by LC × LC [40]. Therefore, the objective of this study was to select complimentary columns in the ^1^D and ^2^D and to choose the best LC × LC method for the separation of a *S. nigra* leaves extract as the most complex sample in this study. Different methods have been optimized and compared regarding the orthogonality, peak capacity, and peak distribution leading to a multi-^2^D approach for an improved separation. Lastly, the multi-^2^D LC × LC method has been applied to various plant parts of the five European herbal remedies and hyphenated to high-resolution mass spectrometry to demonstrate the separation capability based on a chemical characterization of phenolic compounds present in the extracts.

## Material and methods

### Chemicals and materials

Ethanol (> 99.7% (*v/v*)) HPLC grade was purchased from VWR (Darmstadt, Germany). Acetonitrile (100%) was purchased from VWR (Rosny-sous-Bois-cedex, France), and methanol ($\ge$ 99.9% (*v/v*)) from VWR (Leuven, Belgium), both HPLC–MS grade. Formic acid (≥ 99%) was supplied by Fisher Scientific (Schwerte, Germany). Cellulose filters type 15 A with a 110 nm diameter were obtained from Carl Roth (Karlsruhe, Germany) and PTFE filters with 0.20 µm pore size and a diameter of 13 mm from Macherey–Nagel (Düren, Germany). Ultrapure water (resistivity 18.2 M Ω cm^−1^) was daily obtained from an Ultrapure Water System (Sartorius, Goettingen, Germany).

### Sample preparation

Dried flowers (Bosnia, 2021), berries (Poland, 2021), and barks (Serbia, 2020) from *Sambucus nigra* L. were obtained in a drug store (Herbathek, Berlin, Germany). *Sambucus nigra* leaves were collected in Essen, Germany, in 2023, and air-dried at room temperature. Sponsored by Alfred Galke GmbH (Bad Grund, Germany) were dried flowers (Poland, 2022) and berries (Poland, 2020) of *S. nigra*; leaves (Serbia, 2020) of *A. eupatoria*; leaves, seeds, and roots of *A. archangelica* (Poland 2022); and berries (Bulgaria, 2022) and roots (Poland, 2023) of *S. ebulus*. Dried leaves of *A. eupatoria* were obtained from Ttavu Ykrainy (Ukraine, 2023). From Bulgaria in 2024, leaves, stems, and flowers of *A. eupatoria*; flowers and stems from *A. archangelica*; and flowers and leaves of *S. ebulus* were collected and air-dried at room temperature. Whole plants of *A. sylvestris* were purchased in 2024 from Staudengaertnerei Gaissmayer (Illertissen, Germany) and taken apart directly into leaves, stems, and roots and air-dried at room temperature. The plant material was ground to a homogeneous powder. One hundred twenty-five milligrams of ground plant material was extracted using 5 mL of 20, 60, or 90% (*v*/*v*) aqueous ethanol depending on the plant material [40]. Twenty percent (*v*/*v*) aqueous ethanol was used for berries and seeds, 90% for leaves of *A. eupatoria*, and 60% for all other plant material in this study. The samples were extracted using an optimized MAE in a microwave system (Mars NP-1185, Matthews, USA) for 5 min with a microwave power of 400 W at 55 °C [40]. The extracts were stored after filtration at −80 °C.

### LC × LC instrumentation

A 1290 Infinity II LC × LC-DAD-HRMS system consisted of a multisampler module (G7167B), two high speed pumps (G7120A), a MCT oven compartment (G7116B), a DAD detector (G7117B), and an automated controlled 2 positions/4-ports dual valve (G1170A). The LC system was operated and controlled using OpenLab CDS Edition (Agilent, Santa Clara, USA) and hyphenated to an Exactive Orbitrap mass spectrometer (ThermoFisher Scientific, CA, USA) with a heated-electrospray ionization source. The sample with the most complex composition was the leaves extract of *S. nigra* which was used for method development. For all methods, 4 µL was injected at an oven temperature of 50 °C. Gradients and columns changing during selection and method development can be found elsewhere (Sect. “Column selection for the 1D and 2D” and “Final optimized LC × LC and multi-2D LC × LC-HRMS analysis”). Before entering the HRMS, the ^2^D flow rate of 2.0–2.5 mL min^−1^ was split in a ratio of 1:5. The parameters for the Orbitrap were full scan with a mass range of *m*/*z* 100–1700, a resolution of 10,000, AGC target reached at 10^6^, a maximum inject time of 10 ms, a spray voltage of 3.5 kV, capillary, and heater temperature of 320 °C and 350 °C, respectively, sheath gas flow of 25 arbitrary units (au.), auxiliary gas flow of 10 au., and sweep gas flow of 1 au.

For the chemical characterization, the LC × LC system was hyphenated to an Agilent 6560 QTOF equipped with an Agilent Dual Jet Stream source. It was operated in positive mode for a full scan data-dependent MS/HRMS acquisition with a mass range of 100 to 1700 m/*z*. The source parameters were capillary voltage of 3.5 kV, nozzle voltage of 750 V, fragmentor voltage of 400 V, gas temperature of 325 °C, sheath gas temperature of 300 °C, nebulizer at 20 psi, drying gas of 5 L min^−1^, and sheath gas flow of 10 L min^−1^. The data processing and analysis were conducted using MS-Dial (4.9) with a MS1 tolerance of 0.01 Da, a MS2 tolerance of 0.025 Da, and a retention time tolerance of 0.5 min for peak alignment. The minimum peak height for detection of a feature was set to 3000 in amplitude, and the peaks were smoothed with a linear moving average level 3. For compound identification and structural validation, acquired MS/MS data were compared against the spectral database MassBank of North America. The features were positive matches when reaching at least 85% similarity between the acquired MS/HRMS spectra and reference spectra from the database. These features were then annotated as tentative candidates (level 2) according to the Schymanski scale [41].

### Column selection for the ^1^D and ^2^D

For the ^1^D column selection, a Kinetex® PFP (150 × 2.1 mm, 1.7 µm), a Luna® Omega polar C18 (150 × 2.1 mm, 1.6 µm), a Luna® HILIC (150 × 2.1 mm, 2.6 µm), and a Luna® CN (150 × 2.0 mm, 3 µm), all from Phenomenex (Torrance, USA), were tested. After selecting PFP as the best ^1^D column, different ^2^D columns were tested for the LC × LC coupling method. For the ^2^D column selection, the columns Kinetex® C18 (50 × 4.6 mm, 2.6 µm), Kinetex® polar C18 (50 × 4.6 mm, 2.6 µm), Kinetex® C8 (50 × 4.6 mm, 2.6 µm), Luna® C5 (50 × 4.6 mm, 5 µm), Kinetex® Phenyl-hexyl (50 × 3 mm, 2.6 µm), and Kinetex® Biphenyl (50 × 3 mm, 2.6 µm), all from Phenomenex (Torrance, USA), were tested. A HILIC mode was also tested for the ^2^D separation; in particular, a Merck SeQuant® ZIC®-HILIC column (50 × 4.6 mm, 5 µm, Merck, Germany) was used to compare the separation power of this separation mode for polar compounds with a polar C18 column. Among the ^2^D columns, the polar C18, the C8, and the ZIC-HILIC columns were selected and optimized for further comparison.

### Final optimized LC × LC and multi-^2^D LC × LC-HRMS analysis

The optimized PFP ^1^D gradient with a flow rate of 80 µL min^−1^ was 0 min 0% B, 10 min 5% B, 16 min 26% B, 25 min 28% B, 30 min 30% B, 34 min 35% B, 38 min 45% B, 40 min 60% B, and 48 min 95% B until 60 min using water with 0.1% formic acid (A), and methanol with 0.1% formic acid (B) as mobile phases. The ^2^D flow rate was set at 2.5 mL min^−1^ using water with 0.1% formic acid (A), and acetonitrile with 0.1% formic acid (B). The modulation time was 0.75 min with two identical 80-µL loops. For the analysis with polar C18 phase as ^2^D column, a gradient of 0–15 min 0–2% B, 15.5–22.5 min 5–15% B, 23–33 min 10–21% B, 34–47 min 15–30% B, and 47–60 min 40–95% B was used. From 42 to 60 min, 95% B was reached after 0.43 min and held until 0.65 min during modulation. For C8 in the ^2^D, the same gradient as for polar C18 was applied except for the first segment between 0 and 15 min where 5–7% B was used since the C8 is not stable at 100% aqueous conditions. The gradient for ZIC-HILIC measurements was 0–20 min 98–50% B and 20–60 min 98–65% B. From 0 to 20 min, 50% B was reached after 0.42 min and held until 0.65 min during modulation. For the multi-^2^D LC × LC setup, the two ^2^D columns selected for the separation of the compounds were the ZIC-HILIC and the polar C18 columns. An automatic controlled 6-port valve was used for the automatic selection of the ^2^D column along the ^1^D analysis. In this way, from 0 to 20.15 min, the ^2^D separation was done using the ZIC-HILIC column. Then, at 20.15 min, the 6-pot valve changed its position, and the ^2^D separation was completed using the polar C18 column. The orthogonality was manually estimated by the bin-counting method with a bin size of 10. As suggested by Leonhardt et al. [25], additionally to the orthogonality estimated by bins, the distribution of the compounds was investigated by calculating the standard deviation between the actual and ideally equal distribution of the compounds over the ^1^D and ^2^D with a value close to zero being optimal. The peak capacity was calculated as described in Li et al*.* and further indicated in the legend of the respective table [42].

## Results and discussion

### Column selection in the ^1^D and ^2^D

**Fig. 1:**
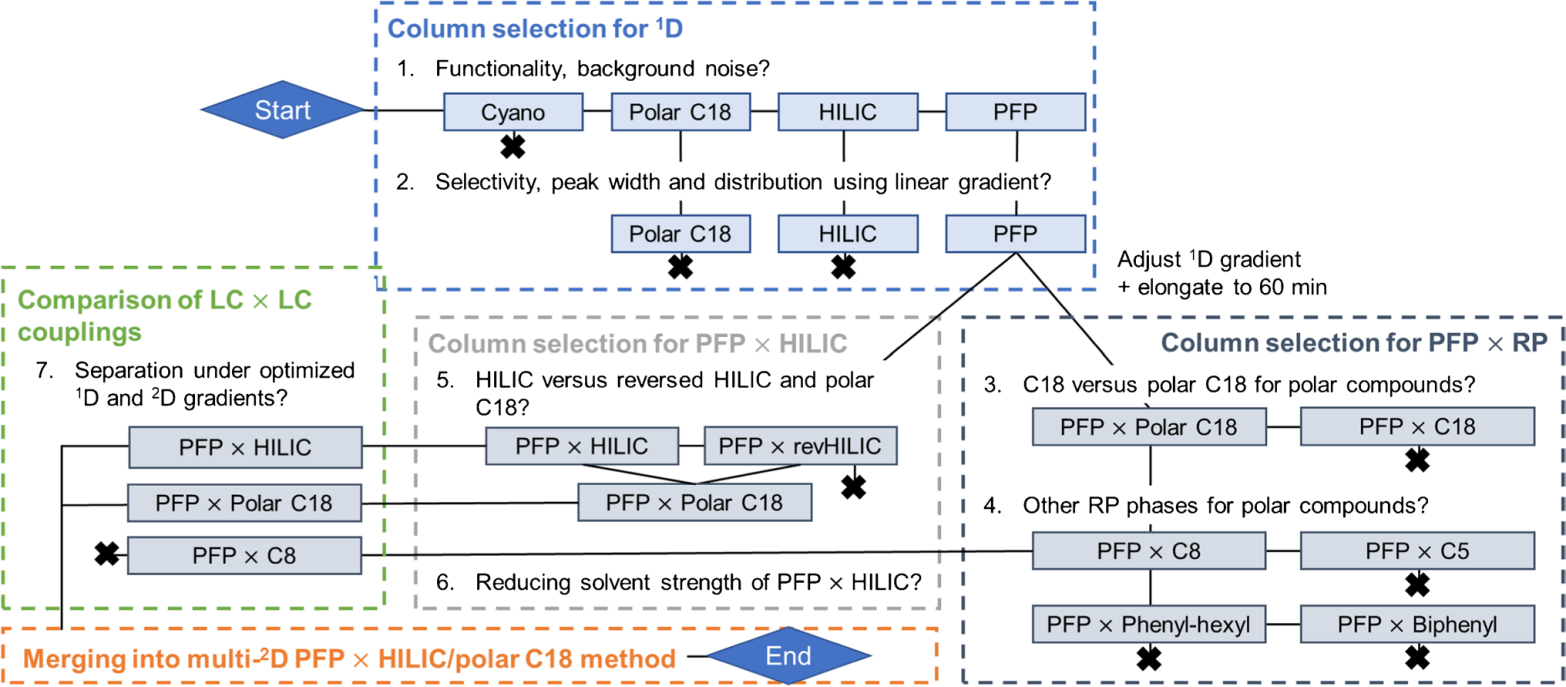
Schematic overview of the process of column screening for the ^1^D and ^2^D, the couplings to LC × LC, the optimization and merging into a multi-^2^D LC × LC method for the analysis of the *S. nigra* leaves extract

Column selection and method development were done to maximize the separation of the *S. nigra* leaves extract which had previously shown the most complex composition out of all five herbal species using a PFP × C18 method developed previously [40]. In Fig. 1, the whole procedure of method optimization is summarized which starts with test measurements of the ^1^D columns for 15 min in order to exclude columns of high background noise or insufficient functionality. The cyano (CN) column showed high background noise and only high abundant compounds while small abundant ones would not be able to be observed (see supplementary Figure S1). The second step involved a linear gradient of 30 min for the PFP, polar C18, and HILIC column to select the best column in terms of selectivity and peak distribution without a time-consuming optimization. The polar C18 column did not provide a good selectivity of high abundant compounds even after optimizing the gradient while especially HILIC lacked completely in terms of retention of phenolic compounds that mostly eluted at the dead time of the setup (see supplementary Figure S1). While polar C18 and HILIC lacked selectivity and good peak distribution, the peak width was noticeably smaller compared to PFP. Broader peak shapes are beneficial for a LC × LC method to avoid the insufficient sampling of the ^1^D peaks known as undersampling which happens if the peaks in the ^1^D are not often enough modulated to maintain the separation of the ^1^D. PFP was therefore chosen as the best ^1^D column due to a high selectivity and peak distribution and the former linear gradient was adjusted and elongated to 60 min by reducing the ^1^D flow rate.

**Fig. 2:**
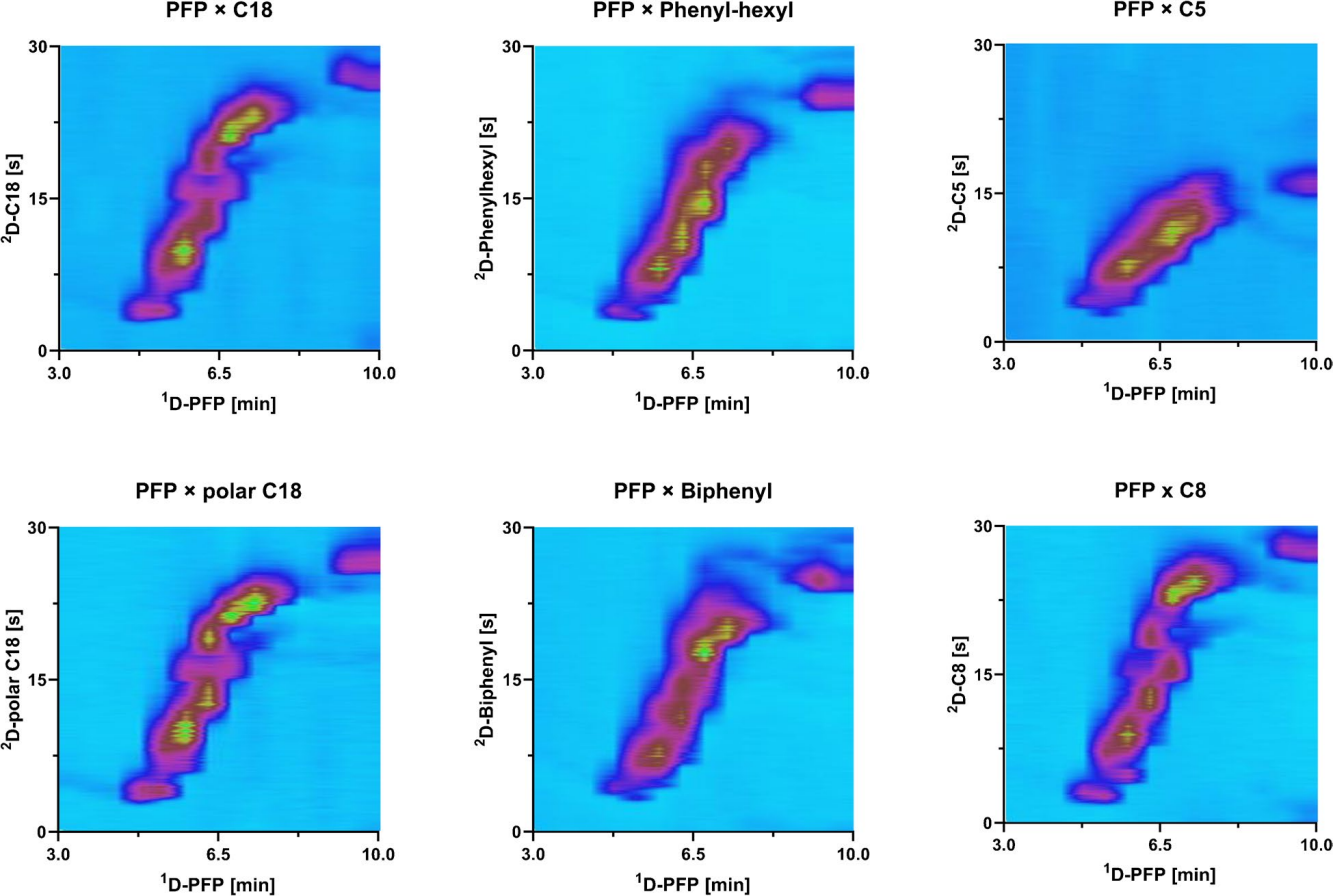
LC × LC-TIC contour plots from 3 to 10 min with different combinations of PFP with C18, polar C18, C5, C8, phenyl-hexyl, and biphenyl, displaying the best-performing ^2^D gradient of 5–7% ACN for C18, polar C18, C5, and C8 and 5–20% ACN for phenyl-hexyl and biphenyl. Additional method parameters: ^1^D flow rate 70 µL min^−1^, ^1^D gradient of 0 min 12% MeOH, 6.5 min 25% MeOH, 19 min 30% MeOH, 35 min 55% MeOH, 42.5 min 95% MeOH until 60 min, ^2^D flow rate 2.5 mL min^−1^, modulation time 0.5 min

In the third and fourth step, the PFP column was coupled to RP phases in the ^2^D. Even though using RP in both dimensions can negatively affect the orthogonality due to correlating separation mechanisms and an insufficient separation of polar compounds, there would be no solvent mismatches making the setup and method development easier [8]. A C18, polar C18, C8, C5, biphenyl, and phenyl-hexyl in RP mode were chosen and tested in the ^2^D to compliment the separation of the PFP in the ^1^D (Fig. 1). The separation of medium to non-polar compounds present in the sample was well separated under RP × RP method developments. This good separation of medium-non-polar phenolic compounds in RP $\times$ RP has been already reported [12, 40, 43]. However, the analysis of samples that contain a mixture of medium and non-polar compounds together with very polar compounds is a big analytical challenge. Therefore, this work on method optimization focused on the complete separation of the compound mixture, including polar compounds such as amino acids and monosaccharides, in order to improve the separation of all compounds contained in the extract. In the third step, the C18 column was compared to the polar C18 for their separation of polar compounds eluting within the first 10 min with a shifting gradient from 0 min 5–7% to 20 min 15–20% ACN. In the fourth step, four other ^2^D columns, i.e., C5, C8, phenyl-hexyl, and biphenyl stationary phase columns, were tested and compared with the C18 columns. While C8 and C5 were chosen due to the reduced hydrophobicity in comparison to C18 stationary phases, biphenyl and phenyl-hexyl columns have proven to provide a different selectivity due to π-π interactions and for biphenyl phases a higher hydrogen bonding capacity than C18 [44, 45]. The optimal shifting gradient for most column started from 5 to 7% ACN, except for the phenyl-hexyl and biphenyl column where it was 5–20% ACN. The direct comparison of the columns with well-performing gradients (Fig. 2) showed that all polar compounds eluted very early and close together in the ^1^D. To avoid coelutions in the ^1^D, the PFP gradient was further optimized before the next step of the optimization (Supplementary Figure S2). Compared to C18 and polar C18 which behaved similarly, the peaks were narrower on the C8 showing better peak resolution and better 2D-space coverage making the C8 a good alternative for further examinations. Biphenyl and phenyl-hexyl had less orthogonality than C18 as well as C5 where the separation seemed to be completely correlated to PFP. The orthogonality was visually categorized, with C8, C18, and polar C18 showing the highest degree of separation, followed by biphenyl and phenyl-hexyl phases, and finally C5 displaying the lowest. Since the polar C18 is out of C8 and C18 the only column being stable at 100% aqueous mobile phase conditions and the separation of polar compounds was favored here, the polar C18 column was chosen for further comparison to the HILIC mode.

**Fig. 3:**
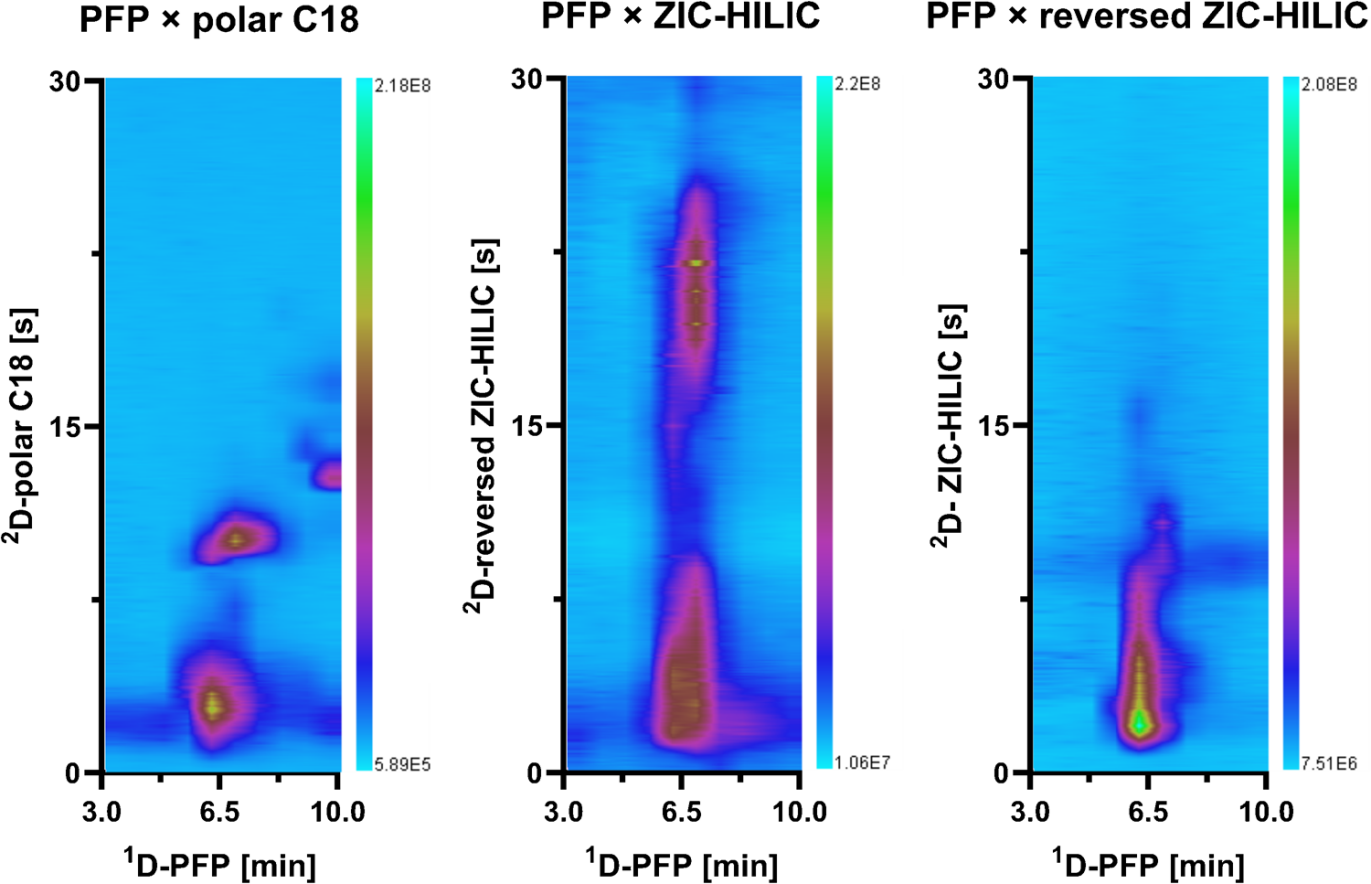
LC × LC-TIC contour plots from 3 to 10 min with combinations of PFP with polar C18 and a gradient of 0–50% ACN in the ^2^D, ZIC-HILIC with 98–50% ACN and reversed ZIC-HILIC with 0–50% ACN. Additional method parameters: ^1^D gradient of 0 min 2% MeOH, 12 min 15% MeOH, 23 min 46% MeOH, 40 min 95% MeOH until 60 min, modulation time 0.75 min, ^2^D flow rate was 2.0 mL min^−1^

The previous results showed that the separation conditions selected for the ^1^D separation produced a strong coelution of the polar compounds hindering more detailed trends; thus, the ^1^D gradient was further optimized to overcome coelutions occurring in the ^1^D (Supplementary Figure S2). With the modified ^1^D gradient, a comparison between RP and HILIC modes was conducted to improve the separation of the polar compounds. In this case, the modulation time was increased to enable more time for the interaction of the injected compounds with the stationary phase while being able to elute all compounds with a full gradient in the ^2^D. When HILIC is coupled into the ^2^D of a LC × LC method with RP in the ^1^D, the water content in the ^1^D gradient highly affects the separation of the ^2^D-HILIC, since water is a strong solvent when using HILIC modes. For this reason, reversed HILIC may be an alternative to improve the solvent strength mismatch. In the fifth step, polar C18 was compared to HILIC mode for the separation of polar compounds. PFP coupled to polar C18 in the ^2^D showed two spots occupied by compounds that were not retained in the ^2^D or having slight retention in the ^2^D (Fig. 3). Under reversed ZIC-HILIC conditions, there was mainly one big spot with little to no retention or a slight retention in the ^2^D for a few compounds. For ZIC-HILIC, the two spots appeared to be even less defined due to different retention times of several compounds but had overall good orthogonality because of the higher interaction with the water layer formed in HILIC modes. To evaluate the whole separation along the complete chromatogram, the EICs of 40 compounds were compared (data not shown). The peak shape of these 40 compounds revealed that the peak width in HILIC mode was significantly higher compared to polar C18. As the ZIC-HILIC separation yielded the best orthogonality of all tested columns, it was considered a good alternative to the polar C18 as ^2^D column. Thus, for the separation of the most polar compounds, the best column in terms of orthogonality was ZIC-HILIC. But in terms of solvent strength in the ^2^D, ZIC-HILIC would be more prone to breakthrough and bad focusing compared to any of the other columns. Thus, beside ZIC-HILIC provided with the highest possible orthogonality to PFP, polar C18 and C8 were also chosen to be further optimized.

### Optimization of LC × LC-HRMS methods and comparison to multi-^2^D LC × LC-HRMS

After the column screening, PFP was selected as ^1^D column, while ZIC-HILIC, C8, and polar C18 were chosen for potential ^2^D columns. As the columns already displayed several advantages and disadvantages, they were screened for their co-use in parallel to one of the other columns. To enable better comparison and a possible combination of several ^2^D columns in a multi-^2^D setup, parameters such as mobile phases, ^2^D flow rate, and modulation time were kept constant for all methods.

#### Optimization of the PFP × HILIC coupling

**Fig. 4:**
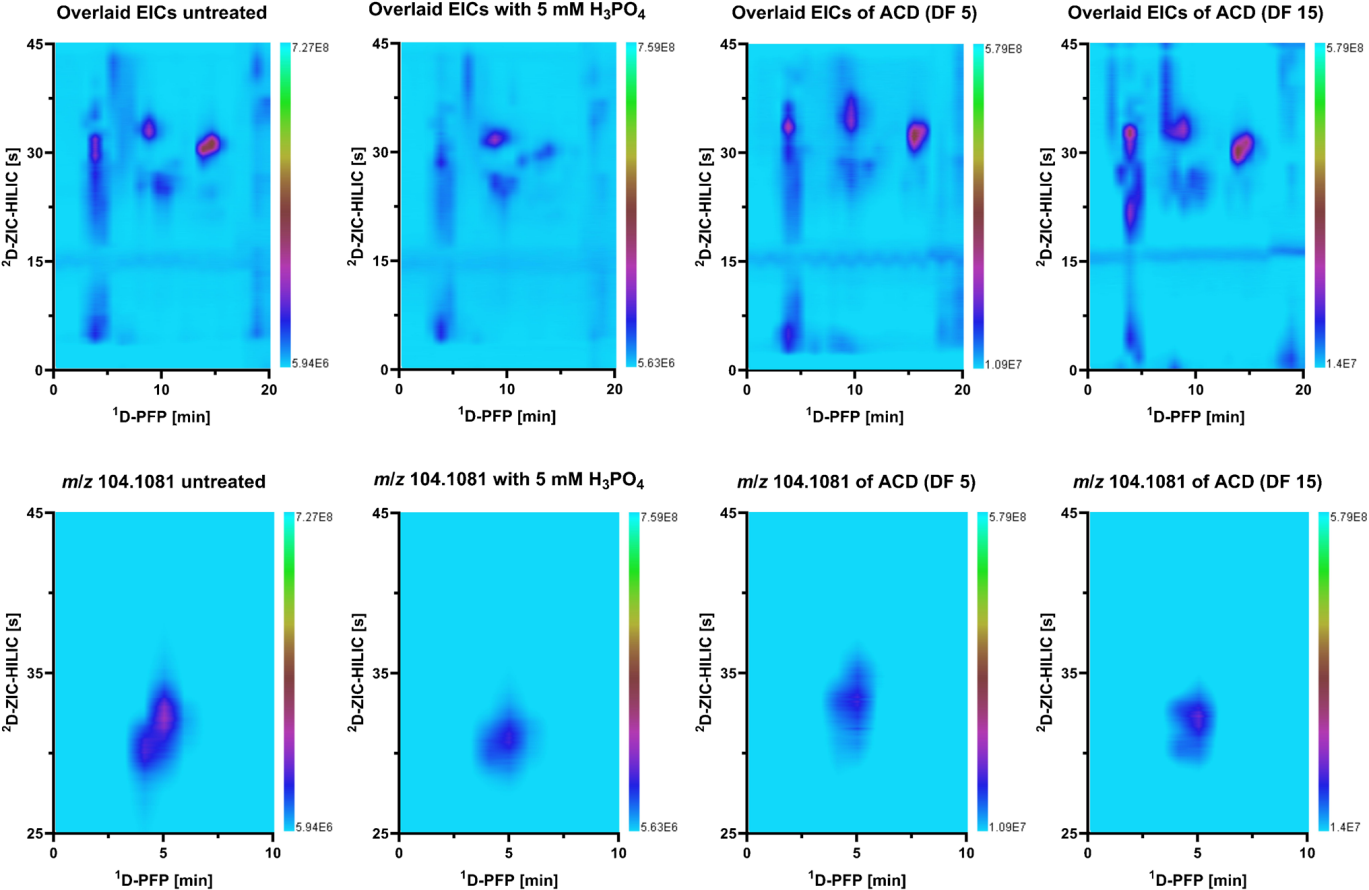
PFP × ZIC-HILIC-HRMS contour plots of overlaid EICs (reduction in background signals by excluding solvent signals from TIC) and the EIC of the *m*/*z* value 104.1081 from 0 to 10 min with no dilution, after addition of 5 mM phosphoric acid to the sample before injection and ACD with dilution factors of 5 and 15

In the case of PFP × HILIC and as sixth step, due to the incompatibility of the solvent strength between the solvents of the ^1^D-RP and ^2^D-HILIC, but the promising orthogonality that this can offer, great efforts were made to improve the separation in the ^2^D-HILIC. Different modulation strategies like passive dilution or at-column dilution (ACD) with different dilution factors (DF) and modifications of the injection solvent and mobile phase such as addition of phosphoric acid or buffers were tested to reduce peak broadening and breakthrough in HILIC mode. For the ACD setup, as described in Chen et al. [16], an additional pump was installed to create a dilution of the ^1^D fractions during the transfer period of the fractions before injection onto the ^2^D. Compared to the conventional setup with passive dilution, referred to as untreated or undiluted, the most remarkable improvements in peak shape were achieved by the addition of 5 mM phosphoric acid to the sample before injection and by setting ACD using a DF of 5 as modulation mode. Figure 4 shows an example of how the different tested conditions affected the retention behavior of several compounds. However, even after this optimization, broader peak widths were observed with the HILIC separation compared to polar C18, which, combined with the presence of coeluting compounds, resulted in not defined signals. The contour plot after addition of phosphoric acid seemed more defined which was confirmed by narrower peaks but also more coelutions of different ions occurred which lead to higher ion suppression about 30–50% in height despite the narrower peaks. The ACD did not improve the separation either. ACD is an effective strategy when the separation in the ^2^D-HILIC presents a pronounced breakthrough, however, in the separation achieved in the developed configuration, a breakthrough was not observable and even the peak widths were similar compared to the undiluted measurement. With increasing ACD dilution factor (from DF 5 to DF 15), the transfer rate from the loop onto the ^2^D column was too slow which caused wrap-arounds, the presence of reoccurring peaks that were not eluted within the modulation time. Since any of the optimization approaches and tests (modulation setups, injection solvent and mobile phase composition) did not result in an overall gain in separation, focusing, or peak width, it was decided to leave the measurements with ZIC-HILIC in the ^2^D under traditional LC × LC instrumental settings in order to reduce the complexity of the system.

#### Optimization of the PFP × RP coupling

**Fig. 5:**
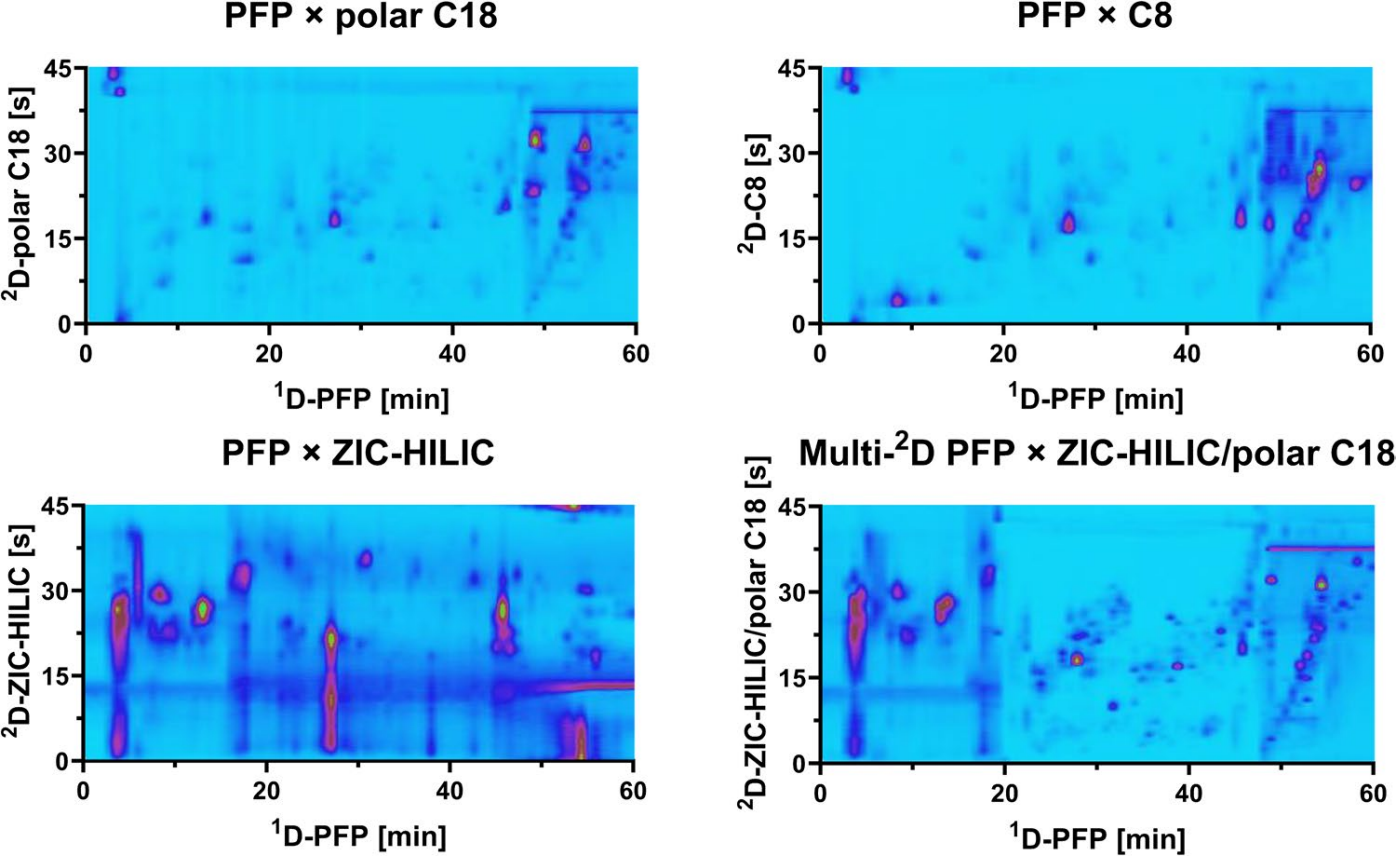
LC × LC-TIC contour plots with combinations of PFP (^1^D) to polar C18, C8, or ZIC-HILIC (^2^D) under optimized method parameters and the multi-^2^D PFP × ZIC-HILIC/polar C18-HRMS contour plot where the ^2^D columns are switched at 20.15 min. Figure was shown previously in LCGC Supplements as Preview for HPLC 2025 [46]

For RP × RP, the best separation was achieved with polar C18 and C8 as ^2^D columns with a gradient of minimal change in the mobile phase at the beginning and middle part (0–47 min) and a nearly full gradient from 47 to 95% ACN with a hold time at 95% ACN towards the end (Supplementary Figure S3). Comparing the separation achieved using the C8 and the polar C18 in the ^2^D, the main difference was observed for the separation of the most polar compounds (eluted from 0 to 20 min in the ^1^D). The polar C18 column allowed a separation of these compounds between 0 and almost 30 s, while the C8 column only allowed the separation of these compounds up to less than 15 s, resulting in a better orthogonality with the polar C18 than with the C8 column at least for the first 20 min. Moreover, in general, along the whole analysis time, the peak widths for polar C18 were narrower than in C8 (Fig. 5), which increased the peak capacity.

#### Optimization of the multi-^2^D LC × LC method

The separation in the ^2^D using ZIC-HILIC enabled the best orthogonality; however, this column also provided the broadest peak widths among all tested columns (Fig. 5 and Table 1) with poor separation of the mid-low polar compounds, and also, the background with this column was higher due to signals arising from the solvent that could potentially mask up other compounds. Otherwise, the ZIC-HILIC column provided the best separation for the most polar compounds. To combine the advantages of the best-performing columns, a multi-^2^D setup was applied where an additional automatic controlled 6-port valve was installed as column selector between different ^2^D columns as described by Montero et al. [43]. The multi-^2^D setup consisted of using the ZIC-HILIC column as ^2^D column from 0 to 20.15 min, where the polar compounds eluted from the ^1^D, and the polar characteristic of the HILIC column provided the best separation performance. The polar C18 column was selected from 20.15 min until the end of the analysis to take advantage of the improved peak capacity offered by this column while also the orthogonality of phenolic compounds eluting between 25 and 45 min was obtained. The resulting multi-^2^D PFP × ZIC-HILIC/polar C18 TIC contour plot showed not only an overall better orthogonality over the individual LC × LC methods but also the tailing of the peaks of the polar C18 separation disappeared (Fig. 5). Additionally, there was a huge improvement of the peak shapes resulting in higher intensities obtained by multi-^2^D LC × LC compared to LC × LC which increased the number of analytes visible in the TIC plots. The improvement in peak shapes for the polar C18 column was observed after applying the multi-^2^D LC × LC method for approx. 4 runs within 1 day and kept stable from this point on as long as the whole sample was not injected onto the C18 column again. Afterwards, the repeatability of the multi-^2^D LC × LC method within 1 day and over three non-consecutive days was determined. The relative standard deviation in retention time did not exceed 0.93% which was in compliance with the most commonly accepted 2.5% limit for LC × LC analysis.

#### Quality parameters of the LC × LC and multi-^2^D LC × LC methods

**Fig. 6:**
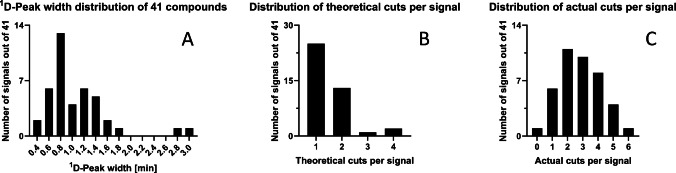
**A** Frequency distributions of peak widths of 41 randomly chosen compounds. **B** Number of theoretical cuts per ^1^D peak. **C** Actual number of cuts per ^1^D peak derived from the EICs of the respective compounds

For the peak capacity of the individual LC × LC and multi-^2^D LC × LC method, 41 compounds were randomly chosen over the whole analysis time and intensity range. An important parameter when developing a LC × LC method is to avoid the undersampling effect. This phenomenon occurs when the sampling time of the ^1^D fractions (modulation time) is too slow and the separation achieved in the ^1^D cannot be maintained during the modulation process. According to theory, peaks should be cut three to four times in order to maintain the separation of the ^1^D and, therefore, avoid undersampling [26, 47]. Because of various structures and functional groups, the distribution of peak widths as well as their theoretical and actual number of cuts per signal during modulation was evaluated. The resulting ^1^D peak widths of the selected 41 compounds were between 0.4 and 3.0 min with a distribution of peak widths that had its maximum (0.8 min) slightly broader than the modulation time (0.75 min) (Fig. 6). When the peak width is smaller than the modulation time (< 0.75 min), the peak will be cut once or not at all. If the peak width is equal or up to twice the modulation time (0.75 to 1.5 min), the peak will be cut twice. To achieve the theoretical 3 to 4 cuts, the peak widths should lie between 1.5 and 2.25 min or 2.25 and 3.0 min, respectively. Applying this, the theoretical number of cuts performed in this LC × LC method was mostly one or two which would imply undersampling due to remixing of compounds before injecting onto the ^2^D. On the other hand, the method parameters were optimized by investigating the actual number of cuts per signal that were derived from the EICs of the compounds. The manual peak width determination may have falsified the results, while the actual number of cuts per signal was counted according to the peak picking algorithm of the software used to control the Orbitrap. Therefore, the distribution of the actual cuts seemed to be more trustworthy and had its maximum at 2 cuts per signals ranging from 0 to 6 cuts due to the concentration variance of the compounds. The mean and median were determined to be 3.8 and 4.0, respectively, which fits the optimal theoretical number of cuts along the ^1^D peak width. Considering that a real sample was analyzed with different compound classes, the method parameters such as ^1^D flow rate, gradient, and modulation time influencing the undersampling were considered adequate based on the actual cuts per signals being observed in the EICs.

To estimate the performance of the individual LC × LC methods and the multi-^2^D method, the peak capacity, orthogonality, and distribution of eluting compounds were determined (Table 1). The median of the peak widths determined at the base points for the PFP × polar C18 method was 0.095 min over the whole analysis time which was comparable to the 0.10 min for the PFP × C8 method. In opposite to that, PFP $\times$ ZIC-HILIC had the highest peak widths with a median of 0.18 min which were slightly narrower in the first 20 min with 0.15 min and slightly broader with 0.20 min for the rest of the analysis. Combining ZIC-HILIC and polar C18 in a multi-^2^D setup led to even better peak widths for both columns with 0.12 for ZIC-HILIC and 0.060 for polar C18 that increased the peak capacity of this method compared to the individual LC × LC methods. The resulting peak capacity, whether theoretical, practical, or corrected peak capacity, was higher for the multi-^2^D method compared to the methods with polar C18, C8, or ZIC-HILIC only. The ZIC-HILIC as ^2^D column reached a corrected peak capacity (^2D^n_c_ corrected, Table 1) of 160, whereas with polar C18 and C8, a corrected peak capacity of 222 and 207, respectively, was reached. The multi-^2^D setup benefitted from the best peak shapes of each separation mode, giving in total narrower peak shapes overall and yielded 424 as corrected peak capacity with a peak generation rate of 7.1 peaks min^−1^. In terms of orthogonality, LC × LC method with polar C18 and C8 ^2^D columns yielded similar results with 68% coverage which was increased to 85% with the PFP × ZIC-HILIC method. As expected, due to the advantages of the combination of ZIC-HILIC and polar C18 columns, the orthogonality of the multi-2D measurement was significantly increased achieving a value of 92%. As the orthogonality describes the coverage but not the distribution of the analytes among the 2D-space, the peak distribution was determined by counting the number of analytes per bin and calculating the standard deviation to an ideal equal distribution of the analytes for the ^1^D and ^2^D. The deviation of peak distribution among the ^1^D was determined to be 14.1 which was caused by the clustering in the beginning and end of the method from 0–20 and 47–60 min. In the ^2^D, polar C18 and C8 had the highest SD value of 13 due to the unresolved polar compounds in the beginning which correlated with the lower orthogonality. ZIC-HILIC yielded a value of 10.2 due to an orthogonal separation but the broader, coeluting peaks were a drawback, while with the multi-^2^D method, it was further reduced to 7.1 due to the advantages of the different ^2^D columns.

Therefore, the multi-^2^D method was superior in terms of peak capacity, orthogonality, and peak distribution compared to each individual LC × LC method. The previously reported multi-^2^D method by Montero et al. [43] had a corrected peak capacity of 1041 (8.7 peaks min^−1^) and 68% orthogonality for the multi-^2^D method. Hurk et al. proved how strongly corrected peak capacity can vary due to the applied ^2^D gradient in RP × RP analysis ranging from 263 (4.4 peaks min^−1^) for a full gradient to 779 (13.0 peaks min^−1^) with a shifted gradient and yielded a similar peak capacity for a parallel gradient in the ^2^D while reaching the highest orthogonality of 74% [9]. The RP × RP analysis of phenolic compounds in grape juice and wine varied from 656 to 1013 (14.6 to 22.5 peak min^−1^) and from 54 to 80% for different samples [48]. Therefore, the performance of LC × LC analysis in terms of peak capacity and orthogonality will strongly be affected by the columns and their separation mechanisms, method parameters such as flow rates and gradients, the sample itself, and the methods used for the determination of such characteristics which makes an internal comparison between methods inevitable. Among recent LC × LC method developments, there is always either the challenge of reaching orthogonality due to similar retention mechanisms or in cases of very different mechanisms, the challenge of combining both dimensions. In the first case, the improvements in orthogonality are limited to changing gradient conditions such as pH, buffer, or proportion of mobile phase [49, 50]. In the second case, additional equipment is often necessary to dilute the solvent eluting from the ^1^D to focus analytes on the head of the column [43, 51, 52]. Even though for all these challenges, there are respective solutions as shift or parallel gradients [10, 11, 53] as well as advanced modulation strategies like ACD [20, 54], but the methods are often not applicable to samples of wide polarity range or without the required additional equipment. In opposite to that, the here presented method combines the advantages of both approaches due to the selection of several stationary phases that are chosen in dependency on the polarity of the analytes eluting from the ^1^D.

**Table 1:** Peak widths, peak capacities, orthogonality, and peak distributions of the optimized LC × LC-HRMS and multi-^2^D LC × LC-HRMS methods. The peak widths were determined at the base points of the EICs for the ^1^D and ^2^D, and the peak distribution is given as standard deviation between an ideal and the actual distribution of peaks among the ^1^D and ^2^D

^2^D column (s)	Polar C18	C8	ZIC-HILIC	ZIC-HILIC/polar C18
^1^D analysis time (^1^t_G_)	60 min	60 min	60 min	60 min
^1^D ${\boldsymbol{w}}$	0.90 min	0.90 min	0.90 min	0.90 min
^1^n_c_	67.7	67.7	67.7	67.7
< β >	1.16	1.16	1.16	1.16
^1^n_c_ corrected	58.7	58.7	58.7	58.7
^2D^ ${\boldsymbol{w}}$ (0–20.15 min)	0.11	0.11	0.15	0.12
^2D^ ${\boldsymbol{w}}$ (20.15–60 min)	0.090	0.10	0.20	0.060
^2D^ ${\boldsymbol{w}}$ (0–60 min)	0.095	0.10	0.18	0.065
Modulation time (^2^t_G_)	0.75 min	0.75 min	0.75 min	0.75 min
^2^n_c_	8.9	8.5	5.2	12.5
^2D^n_c_ theoretical	602	560	350	848
^2D^n_c_ practical	327	310	190	460
^2D^n_c_ corrected	222	207	161	424
Orthogonality (A_O_) (%)	68	67	85	92
^2D^SD	13.0	13.1	10.2	7.1

Median of peak width $(w)$; gradient time (${\mathrm{t}}_{G}$); peak capacity ${n}_{C}$ =$\frac{{\mathbf{t}}_{{\boldsymbol{G}}}}{{\boldsymbol{w}}}$; peak capacity in the ^1^D (^1^n_c_) and ^2^D (^2^n_c_) and in a ^2^D LC system (^2D^n_c_); sampling time (${\mathrm{t}}_{S}$); mean width of ^1^D peaks as standard deviation in time units (σ); correction factor for undersampling$<\upbeta > = \frac{{n}_{C}}{\sqrt{1+0.21(\frac{{t}_{s}}{{\sigma }^{2}})}}$; ^2D^n_c_ theoretical $=$ ^1^n_c_ $\times$ ^2^n_c_; ^2D^n_c_ practical$=\frac{2Dnc theoretical }{<\upbeta >}$; ^2D^n_c_ corrected $=$ ^2D^n_c_ practical $\times$ A_O_; orthogonality A_O_; standard deviation (SD)

### Chemical characterization of European herbal remedies by multi-^2^D LC × LC–MS/HRMS

**Fig. 7:**
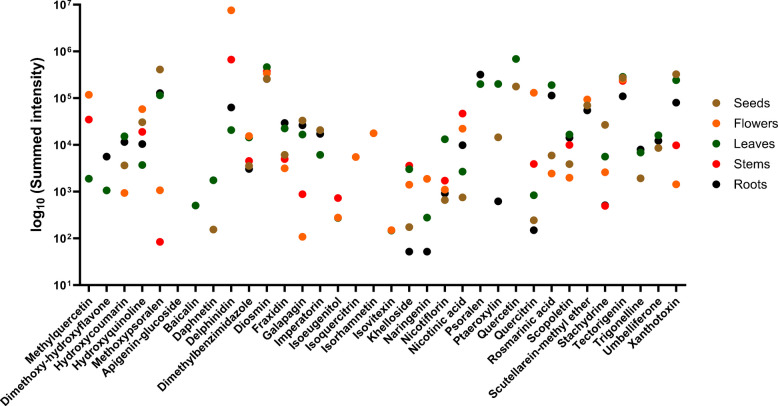
Comparison of the summed intensities of 32 tentative candidates of phenolic compounds present in the flowers, leaves, stems, seeds, and roots of *A. archangelica*. The retention time, precursor and fragment ion, and adduct type are presented in the Supplementary Table S3

Finally, the multi-^2^D LC × LC–MS/MS method was applied to the five European herbal remedies with various plant parts such as flowers, leaves, stems, barks, roots, and seeds (for 2D plots, see supplementary Figure S5). Flowers and leaves were the most complex samples due to the overall number of analytes except for the leaves of *A. eupatoria* from two suppliers. Similarly, roots and barks have the lowest number of analytes where an increased separation as for LC × LC would not be necessary but for the comparison between all samples. The MS/MS spectra were compared with the database MassBank of North America, and features were annotated as tentative candidates level 2 on the Schymanski scale [41] when reaching at least 85% similarity. Features that were repetitive in the feature list due to the modulation of the LC × LC method were combined and the intensities summed up. As the chemical profile of *A. archangelica* was mostly known for the essential oils [36, 55, 56], this plant is described here more in detail than the others. The flowers of *A. archangelica* were rich in methyl quercetin, hydroxyquinoline, delphinidin, isoquercetrin, and isorhamnetin (Fig. 7). Nevertheless, compared to other parts of the plant, the leaves contain the widest range of phenolic compounds, with particularly high concentrations of hydroxycoumarin, quercetin, ptaeroxylin, and nicotiflorin, while they have similar concentrations of diosmin to the flowers and seeds and similar concentrations of xanthoxin to the seeds. The intensities of khelloside and nicotinic acid were the highest in the stems, but also methylquercetin and delphinidin were present in high amounts. Roots had often intensities in the low to mid-range compared to the other parts except for fraxidin and a dimethoxy-hydroxyl flavone, while imperatorin was found primarily in the roots and seeds. Otherwise, the seeds were rich in methoxy psoralen, galapagin, quercetin, stachydrine, and xanthotoxin. The comparative results for the other plant materials in this study are summarized in the Supplementary Figures S6–S9 for *A. eupatoria*, *A. sylvestris*, *S. ebulus*, and *S. nigra, *respectively, and in the Tables S1 and S2 for all plants. For *A. eupatoria*, three suppliers for the leaves resulted in high variance for the phenolic composition while the flower extract often had the lowest intensities. The *Sambucus* species were similar in the distribution and intensities of the phenolic compounds for each plant part with similar ranges for the flowers, leaves, and berries. Some compounds were left out of this comparison in the figures as they only appeared in one plant species; for example, kaempferol-rhamnoside was only found in flowers of *A. archangelica* (Supplementary Table S1). Epicatechin was annotated in all plant parts of *A. eupatoria* with the highest intensity in the flowers but not found in any other plant in this study. Similarly, isovitexin was mostly present in *A. eupatoria* but also found in *A. archangelica* with lower intensities. Lonicerin was present in the flowers and leaves sample of both *Sambucus* species while fraxidin, galapagin, and imperatorin were completely missing in this species (Supplementary Figure S8 and S9). Hence, marker substances can potentially be derived to differentiate between the plant parts and species if necessary for authentication or fraud studies. The number of phenolic compounds and the relatively even distribution among all plants and most of the plant parts in this study highlighted the versatility of their use as alternative medicine. Further studies establishing a direct link between specific phenolic compounds and their effects, as well as synergistic and antagonistic studies, would be helpful in assessing the full potential of these European herbal remedies.

## Conclusion

Comprehensive two-dimensional liquid chromatography is a very powerful analytical technique; however, even so, it has some separation limits, especially for samples with complex mixtures with a wide variety of polarities. In such cases, the use of multi-^2^D LC × LC offers an extra possibility to improve the separation power for analytes depending on their physicochemical properties or if the columns present complementary separation mechanisms or other advantages in general. In this work, among several tested columns for both dimensions, PFP had the best potential for the ^1^D while C8, polar C18, and ZIC-HILIC presented the best potential as ^2^D columns and were, therefore, selected for further optimization. While PFP × polar C18 and PFP × ZIC-HILIC showed both advantages over the others, namely increased peak capacity with polar C18 and improved orthogonality with ZIC-HILIC in the ^2^D, the separation advantages and disadvantages of the individual methods clearly demonstrated that a combination of both would be feasible. The multi-^2^D PFP × ZIC-HILIC/polar C18-HRMS method yielded a higher performance due to the overall higher orthogonality and improved peak capacity with an increase of 91% for the peak capacity, 8.2% for orthogonality, and 30% better peak distribution compared to the best of the individual LC × LC methods. For the chemical characterization of five European herbal remedies, the multi-^2^D LC × LC method was hyphenated to tandem mass spectrometry to evaluate differences in the phenolic profiles of these plants and their plant parts. As the first comparative study including not only these five plants but also several different plant parts, it was possible to observe tendencies between the species with the possibility to establish marker compounds for authentication purposes.

## Supplementary Information

Below is the link to the electronic supplementary material.

Supplementary Material 1 (DOCX 3.40 MB)

## Data Availability

All data are available to share upon request.
