# Supplementary material for: The benefits of multi-2D LC × LC compared to LC × LC for the analysis of European herbal remedies
Source: Anal Bioanal Chem. 2025 Dec 19;418(4):1245–58. doi: 10.1007/s00216-025-06278-0 (PMC12901227; doi:10.1007/s00216-025-06278-0)
Supplement: Supplementary file 1 — Supplementary Material 1 (DOCX 3.40 MB) [file 216_2025_6278_MOESM1_ESM.docx]

**The benefits of multi-^2^D LC** $\boldsymbol{\times}$ **LC compared to LC** $\boldsymbol{\times}$ **LC for the analysis of European herbal remedies**

K. Wetzel^a^, P. Nhan^a^, T. Tishakova^a^, M. Häßler^a^, J.F Ayala-Cabrera^b,c^, L. Montero^d*^, O. J. Schmitz^a*^

^a^Applied Analytical Chemistry, University of Duisburg-Essen, Universitaetsstr. 5, 45141 Essen/Germany

^b^Department of Analytical Chemistry, University of the Basque Country (UPV/EHU), Sarriena Auzoa, 48940 Leioa, Spain

^c^Research Centre for Experimental Marine Biology and Biotechnology, University of the Basque Country (PiE-UPV/EHU), Areatza Hiribidea 47, 48620 Plentzia, Spain

^d^Foodomics Laboratory, Institute of Food Science Research – CIAL (CSIC-UAM), Calle Nicolás

Cabrera 9, 28049 Madrid, Spain

*Corresponding author mail: [oliver.schmitz@uni-due.de](mailto:oliver.schmitz@uni-due.de); [lidia.montero@csic.es](mailto:lidia.montero@csic.es)

**Figures**





**Figure S1**: Base peak chromatograms of the optimized PFP column and the polar C18, HILIC, and CN tested columns after injection of 4 µL of the *S. nigra* extract. The PFP gradient was 0 min 2% MeOH, 7.5 min 10% MeOH, 12 min 46 % MeOH, 18 min 70% MeOH, 20 min 80% MeOH, 25 min 95% MeOH until 30 min at a flow rate of 0.15 mL min^-1^. Polar C18 and HILIC were measured at the same flow rate with gradients of 0 min 2% MeOH, 5 min 5% MeOH, 25 min 95% MeOH until 30 min for polar C18 and 0 min 98% ACN, 5 min 95% ACN, 25 min 50% ACN until 30 min for HILIC. The measurement of the CN column was at a flow rate of 0.3 mL min^-^1 with 0 min 2% MeOH, 2.5 min 5% MeOH, 12.5 min 95% MeOH until 15 min.


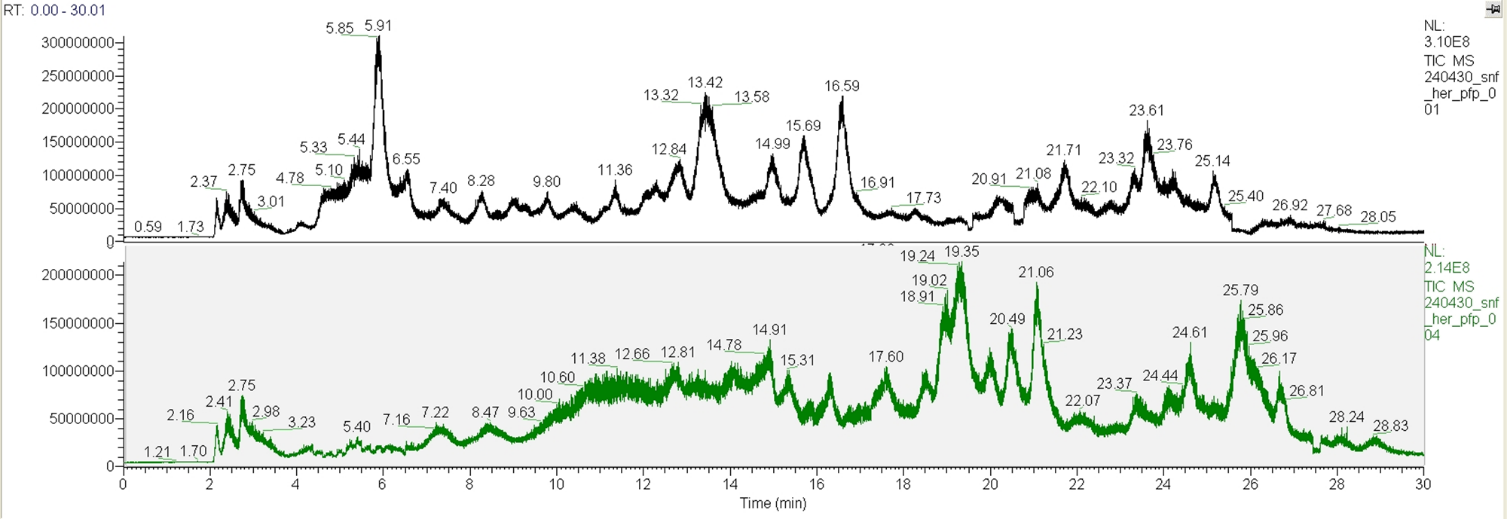


**Figure S2**: TIC plots of PFP gradient before (top) and after modification (bottom). Method parameters for PFP gradient before modification: ^1^D flow rate 150 µL min^-1^, ^1^D gradient of 0 min 12% MeOH, 3.25 min 25% MeOH, 9.5 min 30% MeOH, 17.5 min 55% MeOH, 21.25 min 95% MeOH until 30 min. Gradient after modification: 0 min 0% MeOH, 5 min 5% MeOH, 8 min 26% MeOH, 12.5 min 28% MeOH, 15 min 30% MeOH, 17 min 35% MeOH, 19 min 45% MeOH, 20 min 60% MeOH, 24 min 95% MeOH until 30 min.


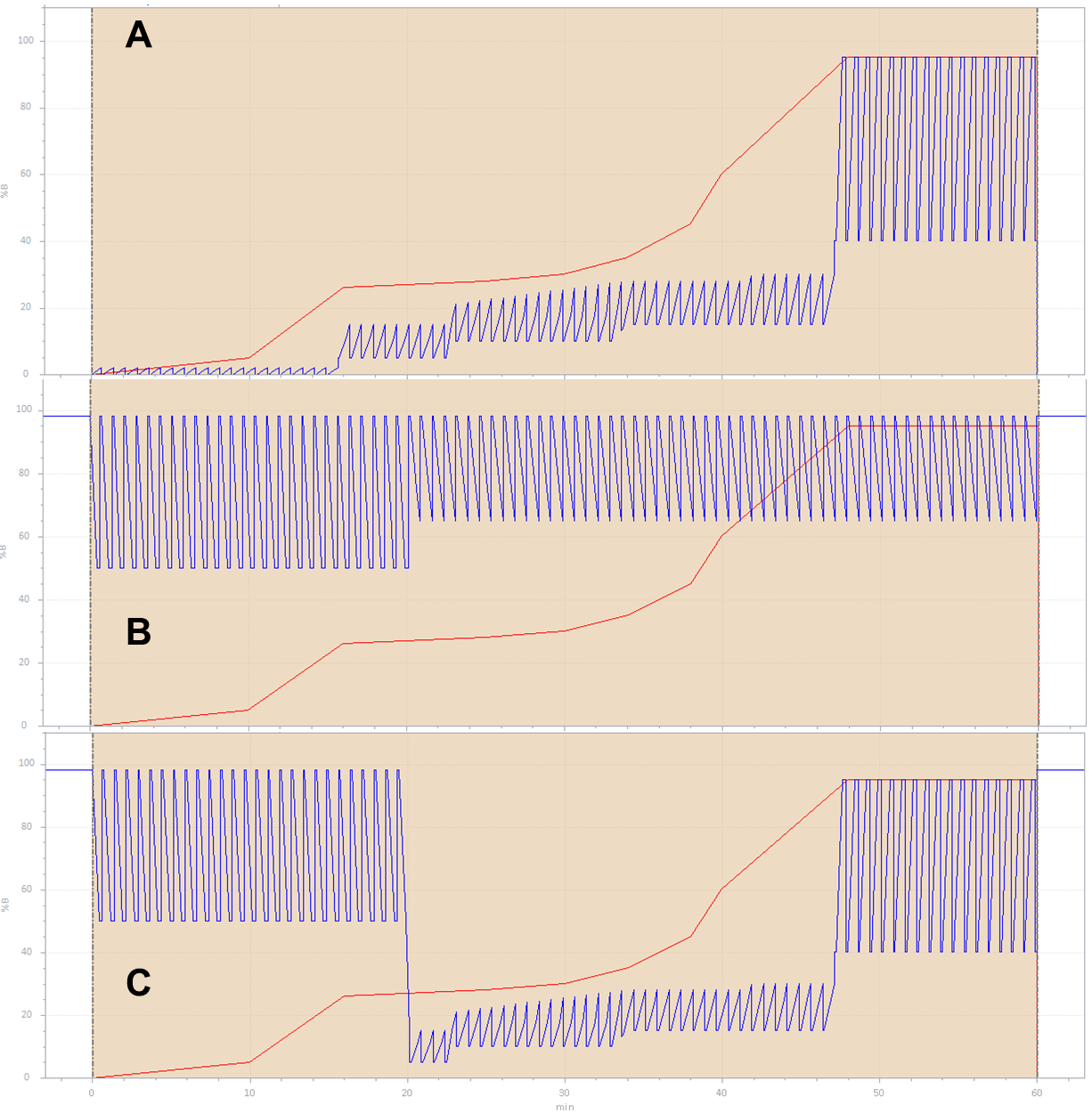


**Figure S3**: LC $\times$ LC gradients in the ^1^D (red) and ^2^D (blue) for the optimized LC $\times$ LC methods. A. Optimal gradient for PFP $\times$ polar C18 measurements. B. Optimal gradient for PFP $\times$ ZIC-HILIC measurements. C. Optimal gradient for multi-^2^D PFP $\times$ ZIC-HILIC/polar C18 measurements.


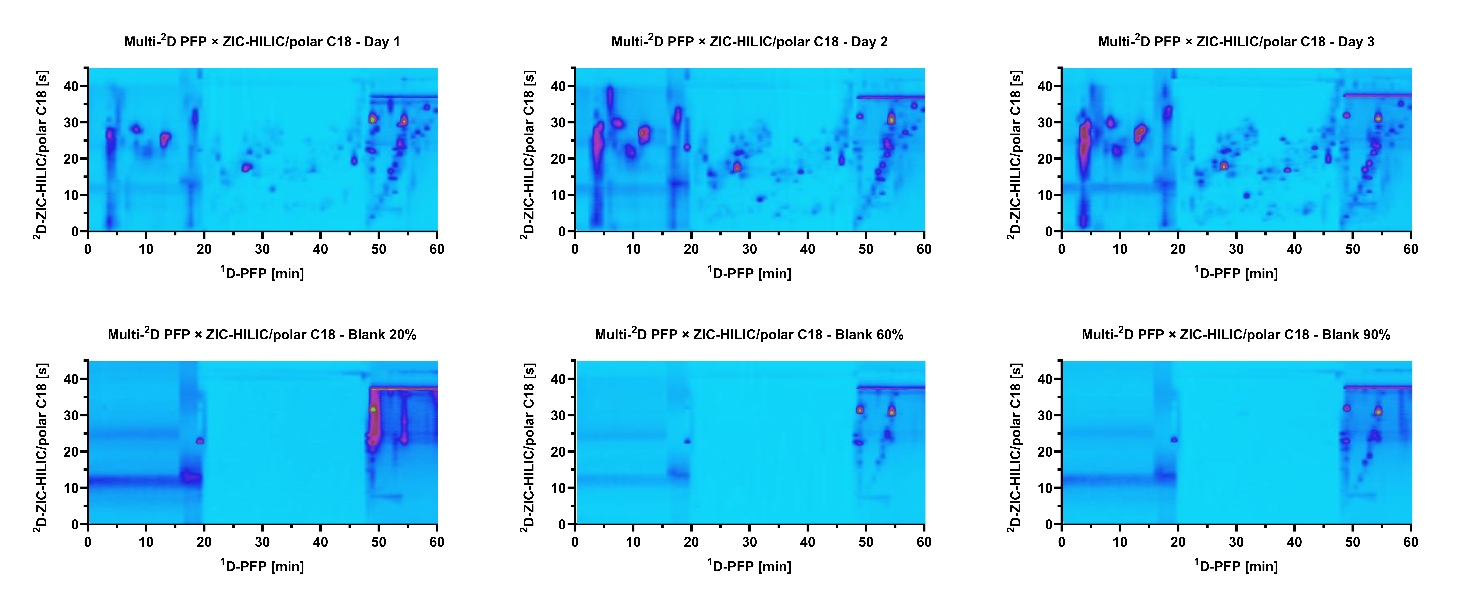


**Figure S4**: On top, the first application and two replicates of following days of the multi-^2^D PFP $\times$ ZIC-HILIC/polar C18-HRMS measurements for the *S. nigra* leaves extract are shown where from day 2 on the results were repeatable. Below, blank measurements are shown analyzed with the same method after injection of the extraction solvents of plant material at 20, 60, and 90% (*v*/*v*) aqueous ethanol after sample preparation.


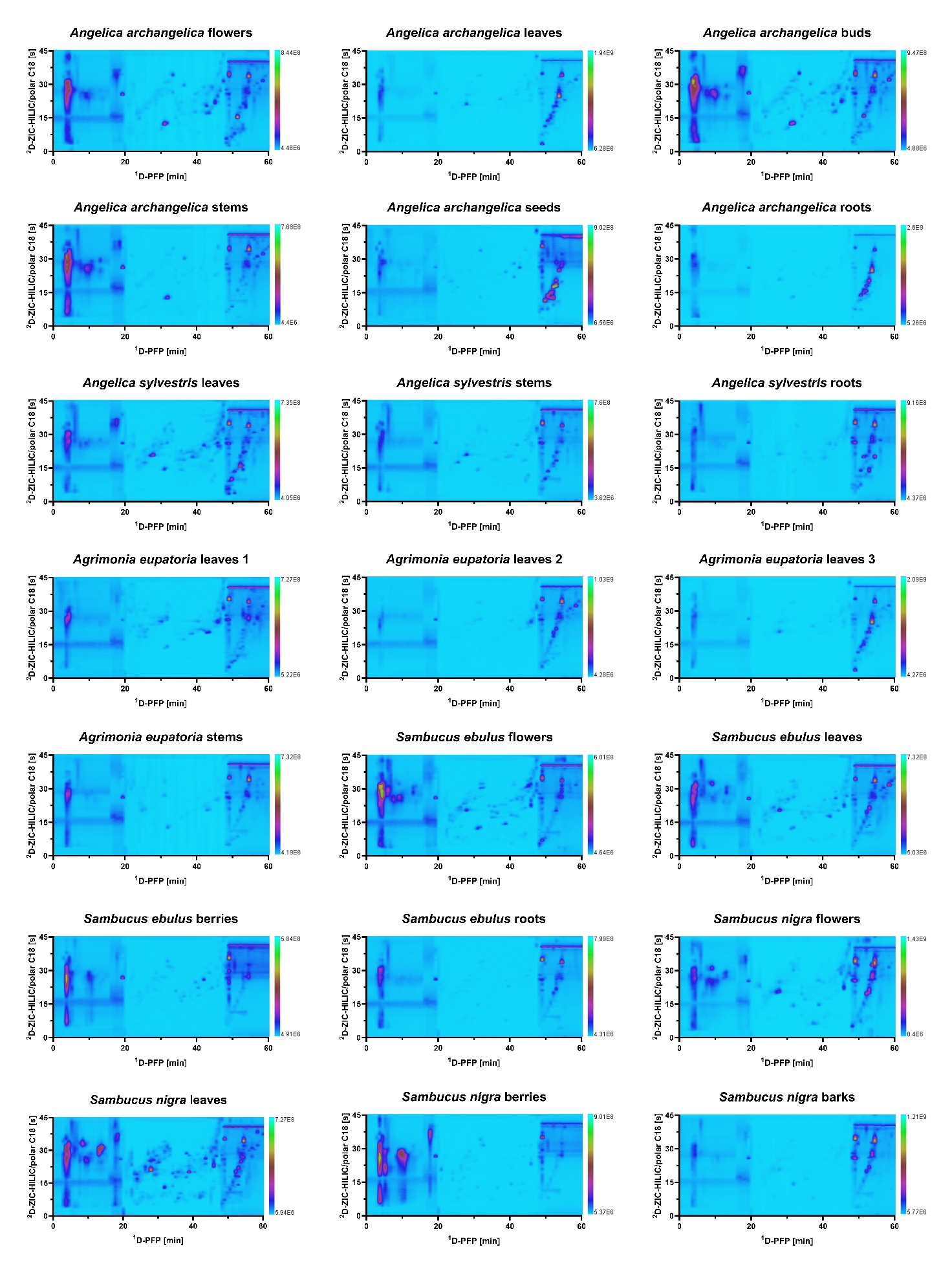


**Figure S5**: 2D TIC plots of the multi-^2^D PFP $\times$ ZIC-HILIC/polar C18-HRMS measurements of all plant material in this study. Samples were measured in between the repeatability measurements of Figure S3 after reaching stable retention times and intensities of the method on day 2.


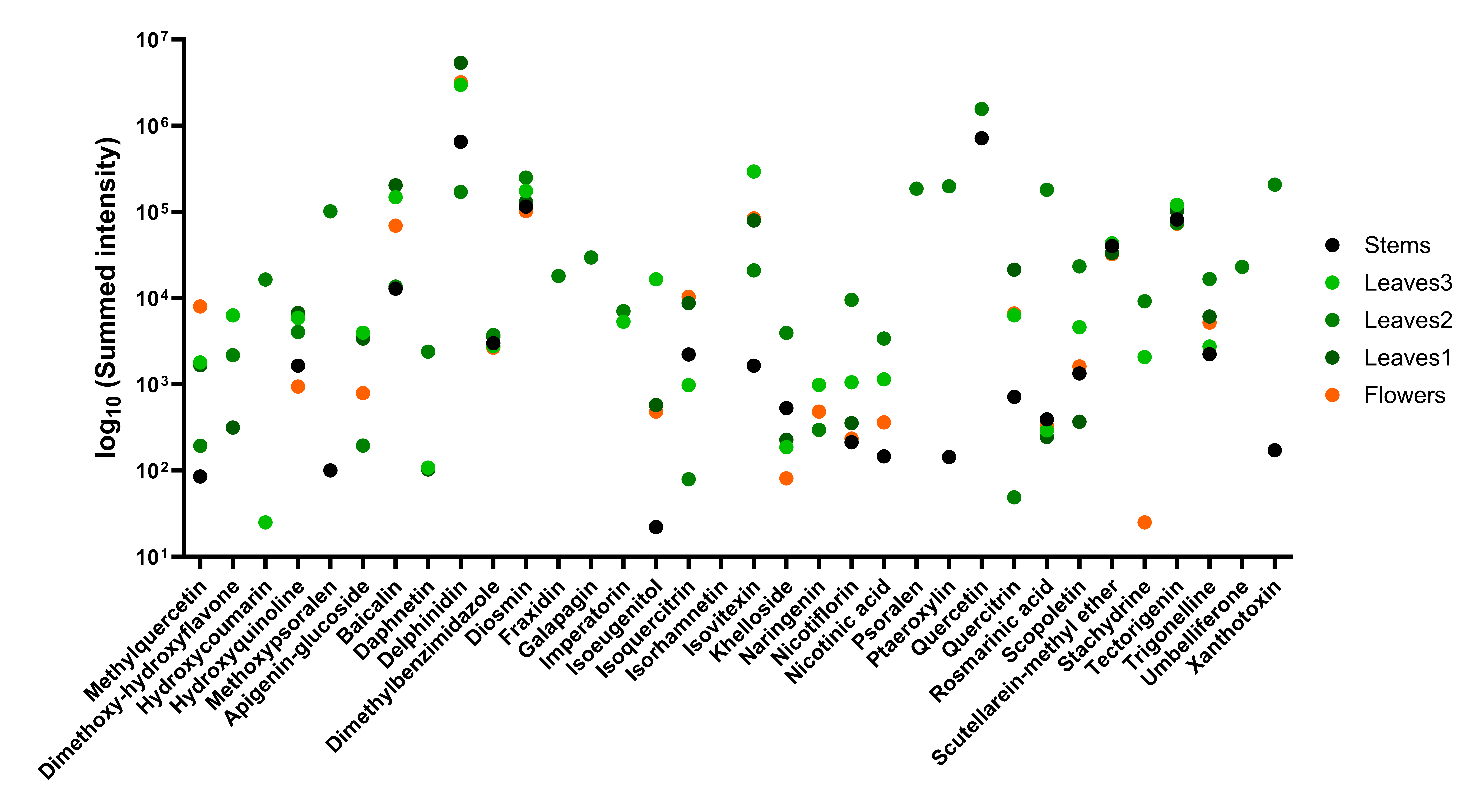


**Figure S6**: Comparison of the summed intensities of 34 tentative candidates of phenolic compounds present in the flowers, leaves obtained from three suppliers, and stems of *A. eupatoria*.


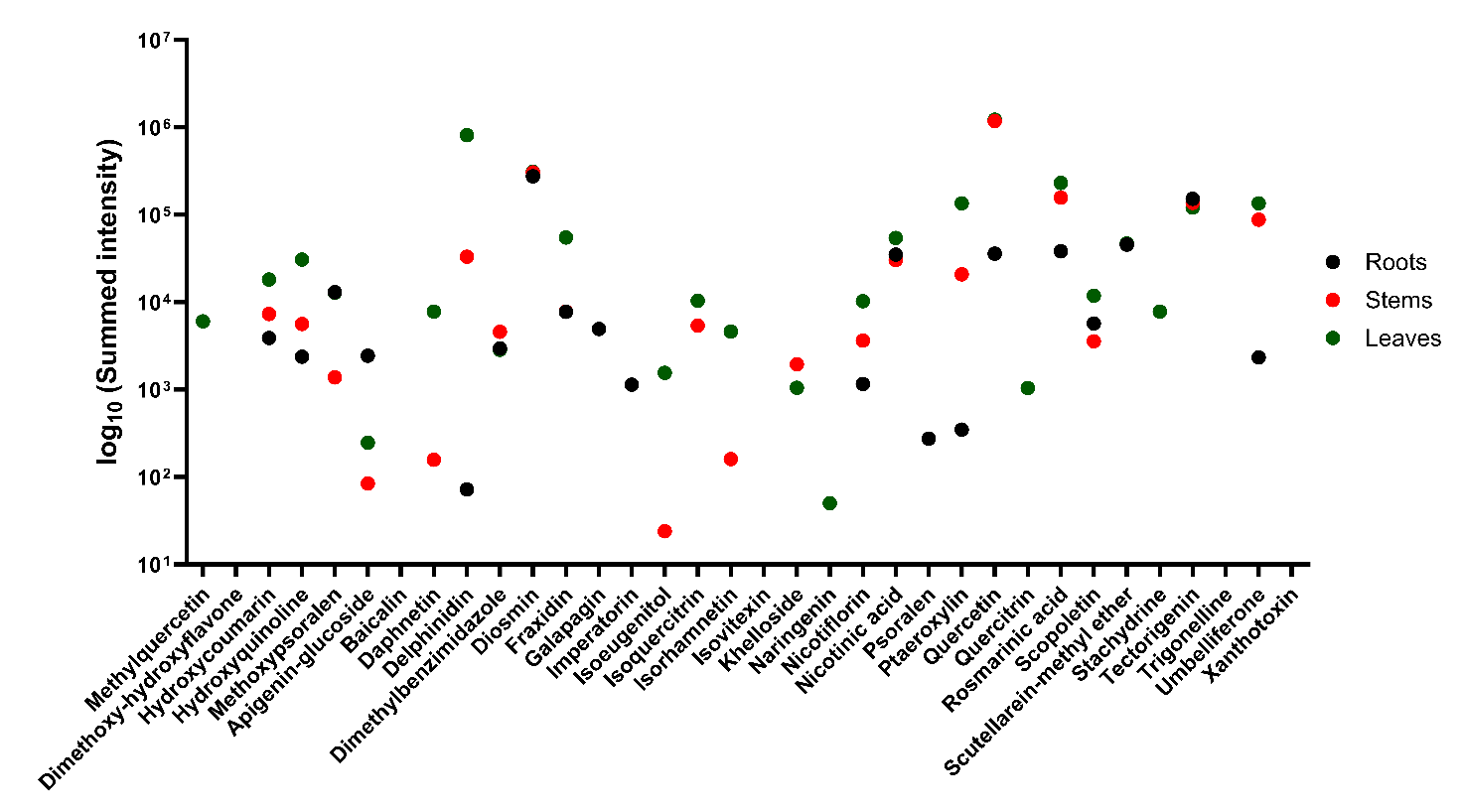


**Figure S7**: Comparison of the summed intensities of 34 tentative candidates of phenolic compounds present in the leaves, stems, and roots of *A. sylvestris*.


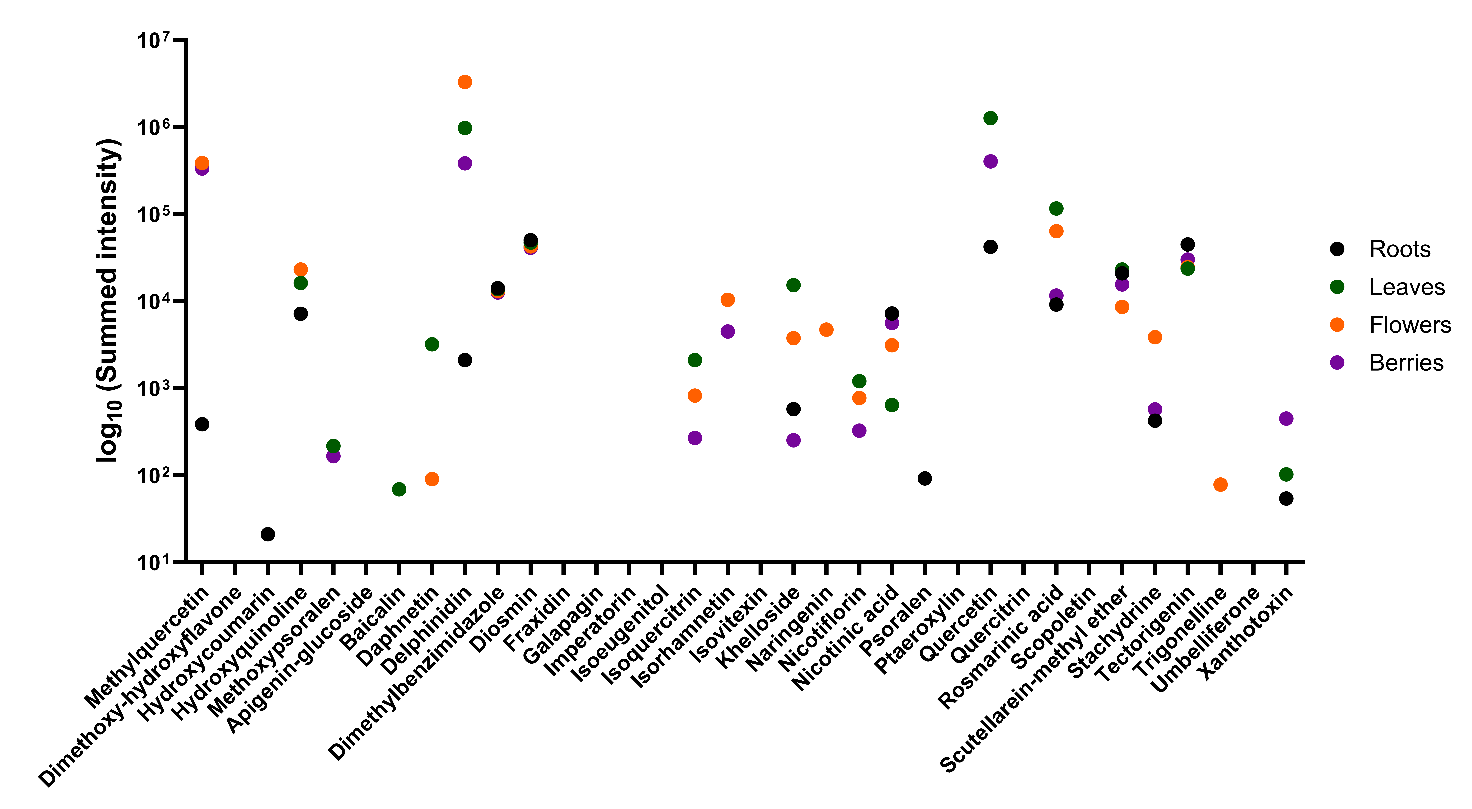


**Figure S8**: Comparison of the summed intensities of 34 tentative candidates of phenolic compounds present in the flowers, leaves, berries, and roots of *S. ebulus*.


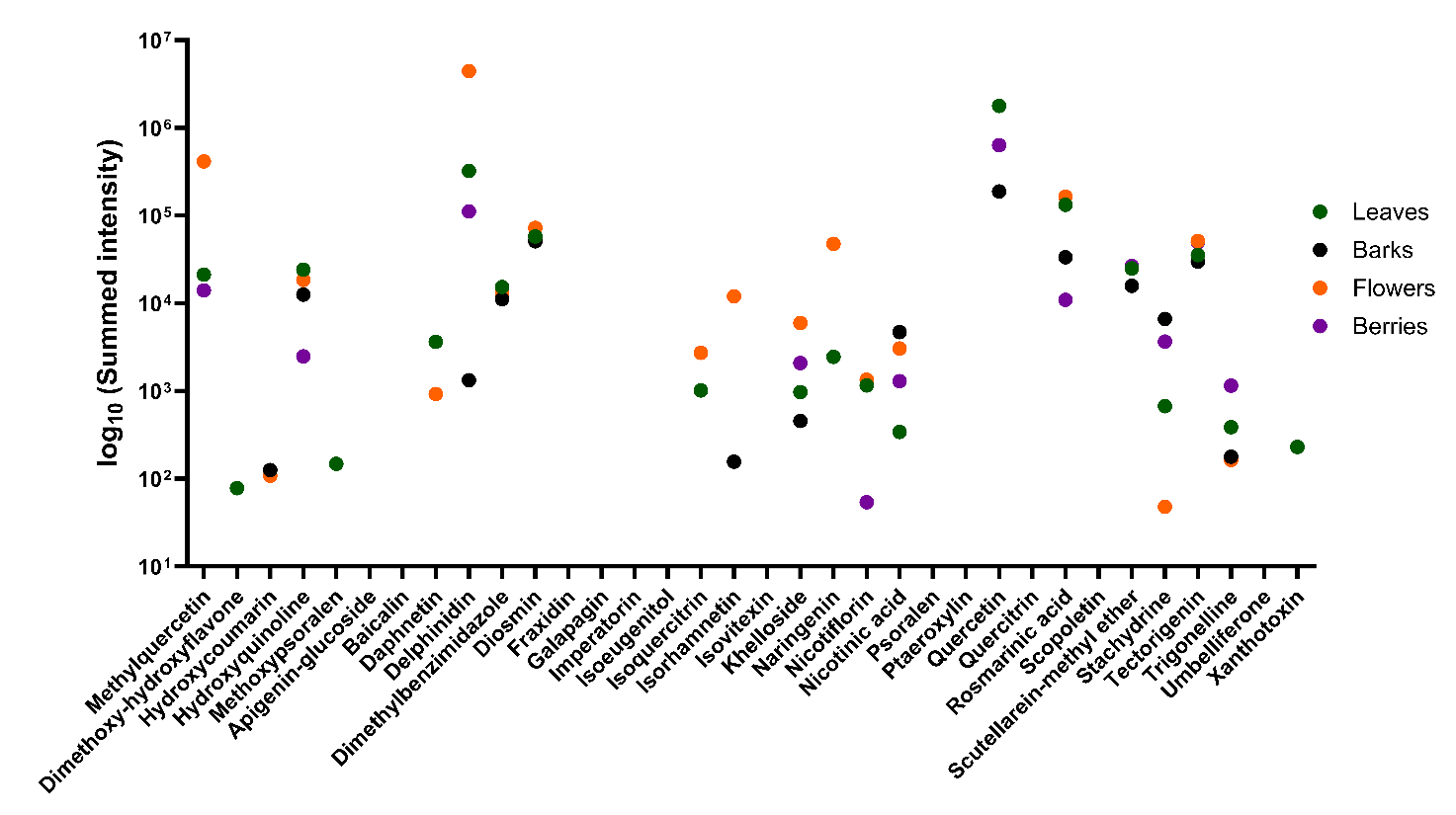


**Figure S9**: Comparison of the summed intensities of 34 tentative candidates of phenolic compounds present in the flowers, leaves, berries, and barks of *S. nigra*.

**Tables**

**Table S1**: Summed intensities of 34 tentative candidates of phenolic compounds present in the various plant parts of *A. archangelica* and *A. eupatoria*.

|  | ***A. archangelica*** | | | | | ***A. eupatoria*** | | | | |
| --- | --- | --- | --- | --- | --- | --- | --- | --- | --- | --- |
|  | **Flowers** | **Leaves** | **Stems** | **Roots** | **Seeds** | **Flowers** | **Leaves1** | **Leaves2** | **Leaves3** | **Stems** |
| **Epicatechin** | 0 | 0 | 0 | 0 | 0 | 18267 | 9303 | 210 | 1155 | 9272 |
| **Methylquercetin** | 118125 | 1886 | 34767 | 0 | 0 | 8017 | 1662 | 194 | 1795 | 85 |
| **Dimethoxy-hydroxyflavone** | 0 | 1061 | 0 | 5609 | 0 | 0 | 315 | 2177 | 6326 | 0 |
| **Hydroxycoumarin** | 936 | 15282 | 0 | 11488 | 3651 | 0 | 0 | 16442 | 25 | 0 |
| **Hydroxyquinoline** | 58210 | 3705 | 18939 | 10406 | 30544 | 945 | 6741 | 4046 | 5909 | 1642 |
| **Methoxypsoralen** | 1070 | 115164 | 84 | 128370 | 410016 | 0 | 0 | 101438 | 0 | 100 |
| **Acetamido-methyl-oxononanoic acid** | 0 | 0 | 0 | 0 | 0 | 0 | 0 | 0 | 0 | 0 |
| **Apigenin-glucoside** | 0 | 0 | 0 | 0 | 0 | 790 | 3398 | 195 | 3963 | 0 |
| **Baicalin** | 0 | 504 | 0 | 0 | 0 | 69527 | 203121 | 13645 | 148098 | 12875 |
| **Daphnetin** | 0 | 1749 | 0 | 0 | 154 | 0 | 103 | 2398 | 108 | 0 |
| **Delphinidin** | 7584662 | 20684 | 667277 | 63105 | 0 | 3196566 | 5352327 | 170651 | 2984191 | 649950 |
| **Delphinidin-galactoside** | 0 | 0 | 0 | 0 | 0 | 4428 | 3553 | 0 | 0 | 441 |
| **Delphinidin-sambubioside** | 0 | 0 | 0 | 0 | 0 | 8561 | 4960 | 0 | 0 | 1834 |
| **Dimethylbenzimidazole** | 15442 | 14426 | 4536 | 3053 | 3561 | 2667 | 3536 | 3733 | 2778 | 2993 |
| **Diosmin** | 337638 | 462621 | 372791 | 255736 | 257702 | 102516 | 130259 | 250440 | 174978 | 115071 |
| **Fraxidin** | 3151 | 22480 | 4965 | 29341 | 6136 | 0 | 0 | 18063 | 0 | 0 |
| **Galapagin** | 108 | 16623 | 882 | 26371 | 33177 | 0 | 0 | 29660 | 0 | 0 |
| **Imperatorin** | 0 | 6117 | 0 | 17222 | 20740 | 0 | 0 | 7084 | 5331 | 0 |
| **Isoeugenitol** | 279 | 273 | 730 | 0 | 0 | 483 | 574 | 0 | 16531 | 22 |
| **Isoquercitrin** | 5486 | 0 | 0 | 0 | 0 | 10367 | 8749 | 79 | 981 | 2219 |
| **Isorhamnetin** | 17761 | 0 | 0 | 0 | 0 | 0 | 0 | 0 | 0 | 0 |
| **Isovitexin** | 150 | 147 | 0 | 0 | 0 | 84362 | 79353 | 21015 | 294607 | 1648 |
| **Kaempferol-3-O-rhamnoside** | 16385 | 0 | 0 | 0 | 0 | 0 | 0 | 0 | 0 | 0 |
| **Khelloside** | 1408 | 3008 | 3590 | 52 | 174 | 81 | 227 | 3945 | 186 | 531 |
| **Lonicerin** | 0 | 0 | 0 | 0 | 0 | 0 | 0 | 0 | 0 | 0 |
| **Lupanine** | 0 | 0 | 0 | 0 | 0 | 0 | 0 | 0 | 7180 | 0 |
| **Naringenin** | 1877 | 278 | 0 | 52 | 0 | 482 | 0 | 297 | 986 | 0 |
| **Nicotiflorin** | 1105 | 13200 | 1717 | 918 | 659 | 235 | 355 | 9555 | 1052 | 212 |
| **Nicotinic acid** | 22273 | 2680 | 46845 | 9865 | 751 | 361 | 0 | 3406 | 1147 | 146 |
| **Orsellinic acid** | 94 | 315 | 0 | 0 | 0 | 572 | 310 | 802 | 0 | 212 |
| **Psoralen** | 0 | 199545 | 0 | 320155 | 0 | 0 | 0 | 186478 | 0 | 0 |
| **Ptaeroxylin** | 0 | 200562 | 0 | 619 | 14531 | 0 | 0 | 198204 | 0 | 143 |
| **Quercetin** | 0 | 687713 | 0 | 0 | 177155 | 0 | 0 | 1564472 | 0 | 715600 |
| **Quercitrin** | 130344 | 838 | 3908 | 150 | 244 | 6629 | 21343 | 49 | 6322 | 715 |
| **Rosmarinic acid** | 2444 | 189693 | 0 | 113921 | 5935 | 330 | 246 | 180980 | 293 | 392 |
| **Scopoletin** | 1984 | 16651 | 9974 | 14226 | 3871 | 1616 | 368 | 23339 | 4601 | 1334 |
| **Scutellarein-methyl ether** | 93663 | 0 | 0 | 54523 | 69698 | 32277 | 33386 | 0 | 43266 | 40284 |
| **Stachydrine** | 2591 | 5611 | 494 | 510 | 26823 | 25 | 0 | 9166 | 2073 | 0 |
| **Tectorigenin** | 248400 | 285433 | 233962 | 109312 | 273258 | 72900 | 103109 | 73828 | 121161 | 81482 |
| **Trigonelline** | 0 | 6849 | 0 | 7946 | 1925 | 5216 | 6126 | 16659 | 2742 | 2232 |
| **Umbelliferone** | 0 | 16084 | 0 | 12270 | 8571 | 0 | 0 | 23001 | 0 | 0 |
| **Xanthotoxin** | 1433 | 241638 | 9759 | 80315 | 324333 | 0 | 0 | 207193 | 0 | 172 |
| **Epicatechin** | 0 | 0 | 0 | 0 | 0 | 18267 | 9303 | 210 | 1155 | 9272 |
| **Methylquercetin** | 118125 | 1886 | 34767 | 0 | 0 | 8017 | 1662 | 194 | 1795 | 85 |
| **Dimethoxy-hydroxyflavone** | 0 | 1061 | 0 | 5609 | 0 | 0 | 315 | 2177 | 6326 | 0 |
| **Hydroxycoumarin** | 936 | 15282 | 0 | 11488 | 3651 | 0 | 0 | 16442 | 25 | 0 |

**Table S2**: Summed intensities of 32 tentative candidates of phenolic compounds present in the various plant parts of *A. sylvestris*, *S. ebulus*, and *S. nigra*.

|  | ***A. sylvestris*** | | | ***S. ebulus*** | | | | ***S. nigra*** | | | |
| --- | --- | --- | --- | --- | --- | --- | --- | --- | --- | --- | --- |
|  | **Leaves** | **Stems** | **Roots** | **Berries** | **Flowers** | **Leaves** | **Roots** | **Berries** | **Flowers** | **Barks** | **Leaves** |
| **Epicatechin** | 0 | 0 | 0 | 0 | 0 | 0 | 0 | 0 | 0 | 227 | 0 |
| **Methylquercetin** | 6044 | 0 | 0 | 333942 | 386248 | 0 | 386 | 14094 | 415954 | 0 | 21305 |
| **Dimethoxy-hydroxyflavone** | 0 | 0 | 0 | 0 | 0 | 0 | 0 | 0 | 0 | 0 | 78 |
| **Hydroxycoumarin** | 18175 | 7355 | 3903 | 0 | 0 | 0 | 21 | 0 | 108 | 126 | 0 |
| **Hydroxyquinoline** | 30765 | 5658 | 2375 | 0 | 23060 | 16187 | 7150 | 2487 | 18576 | 12533 | 24243 |
| **Methoxypsoralen** | 12920 | 1379 | 13047 | 166 | 0 | 216 | 0 | 0 | 0 | 0 | 148 |
| **Acetamido-methyl-oxononanoic acid** | 0 | 0 | 0 | 22683 | 0 | 0 | 16291 | 662 | 0 | 0 | 0 |
| **Apigenin-glucoside** | 247 | 84 | 2437 | 0 | 0 | 0 | 0 | 0 | 0 | 0 | 0 |
| **Baicalin** | 0 | 0 | 0 | 0 | 0 | 69 | 0 | 0 | 0 | 0 | 0 |
| **Daphnetin** | 7814 | 157 | 0 | 0 | 90 | 3201 | 0 | 0 | 923 | 0 | 3631 |
| **Delphinidin** | 815072 | 33146 | 72 | 383830 | 3300920 | 972086 | 2104 | 111818 | 4451766 | 1327 | 323837 |
| **Delphinidin-galactoside** | 835 | 51 | 0 | 0 | 0 | 0 | 0 | 0 | 0 | 0 | 0 |
| **Delphinidin-sambubioside** | 0 | 0 | 0 | 0 | 0 | 0 | 0 | 0 | 0 | 0 | 0 |
| **Dimethylbenzimidazole** | 2851 | 4601 | 2970 | 12574 | 13031 | 13740 | 14071 | 14915 | 13141 | 11165 | 15374 |
| **Diosmin** | 310719 | 306183 | 276962 | 40976 | 42647 | 47023 | 50045 | 58128 | 72290 | 51050 | 57874 |
| **Fraxidin** | 54871 | 7818 | 7750 | 0 | 0 | 0 | 0 | 0 | 0 | 0 | 0 |
| **Galapagin** | 0 | 0 | 4940 | 0 | 0 | 0 | 0 | 0 | 0 | 0 | 0 |
| **Imperatorin** | 0 | 0 | 1137 | 0 | 0 | 0 | 0 | 0 | 0 | 0 | 0 |
| **Isoeugenitol** | 1554 | 24 | 0 | 0 | 0 | 0 | 0 | 0 | 0 | 0 | 0 |
| **Isoquercitrin** | 10405 | 5408 | 0 | 268 | 820 | 2100 | 0 | 0 | 2738 | 0 | 1013 |
| **Isorhamnetin** | 4606 | 160 | 0 | 4480 | 10370 | 0 | 0 | 0 | 11998 | 156 | 0 |
| **Isovitexin** | 0 | 0 | 0 | 0 | 0 | 0 | 0 | 0 | 0 | 0 | 0 |
| **Kaempferol-3-O-rhamnoside** | 0 | 0 | 0 | 0 | 0 | 0 | 0 | 0 | 0 | 0 | 0 |
| **Khelloside** | 1051 | 1944 | 0 | 252 | 3749 | 15262 | 573 | 2083 | 5955 | 455 | 971 |
| **Lonicerin** | 27 | 90 | 0 | 0 | 2419 | 3653 | 0 | 0 | 3923 | 0 | 6712 |
| **Lupanine** | 0 | 0 | 0 | 0 | 0 | 0 | 0 | 0 | 0 | 0 | 0 |
| **Naringenin** | 50 | 0 | 0 | 0 | 4709 | 0 | 0 | 0 | 47664 | 0 | 2452 |
| **Nicotiflorin** | 10265 | 3645 | 1160 | 326 | 770 | 1202 | 0 | 54 | 1350 | 0 | 1159 |
| **Nicotinic acid** | 54264 | 30369 | 35044 | 5595 | 3120 | 641 | 7193 | 1299 | 3058 | 4703 | 341 |
| **Orsellinic acid** | 4788 | 1481 | 0 | 0 | 0 | 0 | 0 | 0 | 96 | 0 | 0 |
| **Psoralen** | 0 | 0 | 275 | 0 | 0 | 0 | 92 | 0 | 0 | 0 | 0 |
| **Ptaeroxylin** | 135418 | 20802 | 348 | 0 | 0 | 0 | 0 | 0 | 0 | 0 | 0 |
| **Quercetin** | 1223083 | 1178040 | 35939 | 401673 | 0 | 1263809 | 42086 | 634551 | 0 | 188766 | 1779045 |
| **Quercitrin** | 1044 | 0 | 0 | 0 | 0 | 0 | 0 | 0 | 0 | 0 | 0 |
| **Rosmarinic acid** | 231724 | 157738 | 38374 | 11552 | 63434 | 116049 | 9139 | 10946 | 164628 | 33602 | 132742 |
| **Scopoletin** | 11894 | 3563 | 5700 | 0 | 0 | 0 | 0 | 0 | 0 | 0 | 0 |
| **Scutellarein-methyl ether** | 47049 | 0 | 45623 | 15542 | 8563 | 23091 | 20854 | 26619 | 0 | 15805 | 25075 |
| **Stachydrine** | 7800 | 0 | 0 | 571 | 3852 | 0 | 422 | 3668 | 48 | 6644 | 673 |
| **Tectorigenin** | 121259 | 137239 | 152343 | 29942 | 24508 | 23685 | 44725 | 49756 | 51238 | 29908 | 35529 |
| **Trigonelline** | 0 | 0 | 0 | 0 | 78 | 0 | 0 | 1149 | 163 | 178 | 387 |
| **Umbelliferone** | 135650 | 87757 | 2328 | 0 | 0 | 0 | 0 | 0 | 0 | 0 | 0 |
| **Xanthotoxin** | 0 | 0 | 0 | 447 | 0 | 102 | 54 | 0 | 0 | 0 | 231 |
| **Epicatechin** | 0 | 0 | 0 | 0 | 0 | 0 | 0 | 0 | 0 | 227 | 0 |
| **Methylquercetin** | 6044 | 0 | 0 | 333942 | 386248 | 0 | 386 | 14094 | 415954 | 0 | 21305 |
| **Dimethoxy-hydroxyflavone** | 0 | 0 | 0 | 0 | 0 | 0 | 0 | 0 | 0 | 0 | 78 |
| **Hydroxycoumarin** | 18175 | 7355 | 3903 | 0 | 0 | 0 | 21 | 0 | 108 | 126 | 0 |

**Table S3**: Overview of the retention time, precursor and fragmentor ions (mass error of 5 ppm), the adduct type, and formula of 32 tentative candidates of phenolic compounds annotated in various plant parts of *A. sylvestris*, *S. ebulus*, and *S. nigra*.

| **Retention time [min]** | **Precursor *m*/*z*** | **Adduct type** | **Formula** | **MS/MS spectrum** | **Reference m/z** | **Annotation** |
| --- | --- | --- | --- | --- | --- | --- |
| 6.04 | 124.0365 | [M+H]+ | C6H5NO2 | 124.036:4955 | 124.0394 | Nicotinic acid |
| 18.397 | 266.13525 | [M+Na]+ | C12H21NO4 | 266.12958:220 | 266.13626 | Acetamido-methyl-oxononanoic acid |
| 20.854 | 146.05995 | [M+H]+ | C9H7NO | 146.05995:412 | 146.06004 | Hydroxyquinoline |
| 21.548 | 223.05794 | [M+H]+ | C11H10O5 | 223.058:4466 | 223.0601 | Fraxidin |
| 24.687 | 179.03476 | [M+H]+ | C9H6O4 | 179.03011:223 | 179.03391 | Daphnetin |
| 25.531 | 291.0871 | [M+H]+ | C15H14O6 | 123.043:1838 139.033:1309 147.041:983 165.052:300 | 291.08682 | Epicatechin |
| 27.036 | 193.04953 | [M+H]+ | C10H8O4 | 133.02777:71 178.02606:57 193.04953:262 | 193.04961 | Scopoletin |
| 27.109 | 303.04883 | [M]+ | C15H11O7 | 303.04901:24487 | 303.04938 | Delphinidin |
| 29.292 | 465.09827 | [M+H]+ | C21H20O12 | 303.04901:7785 | 465.10001 | Isoquercitrin |
| 29.68 | 249.19456 | [M+H]+ | C15H24N2O | 249.19501:3153 | 249.19611 | Lupanine |
| 30.819 | 409.10742 | [M+H]+ | C19H20O10 | 247.052:516 409.09299:278 409.10699:4249 | 409.1123 | Khelloside |
| 32.162 | 169.04884 | [M+H]+ | C8H8O4 | 151.0377:53 | 169.05 | Orsellinic acid |
| 35.774 | 317.06387 | [M+H]+ | C16H12O7 | 302.03799:768 317.05099:1602 317.064:14077 | 317.065 | Methylquercetin |
| 35.774 | 317.06387 | [M+H]+ | C16H12O7 | 317.05148:50 317.06387:334 | 317.06558 | Isorhamnetin |
| 36.646 | 447.12631 | [M+Na]+ | C20H24O10 | 447.12631:280 | 447.12701 | Galapagin |
| 37.39 | 187.04039 | [M+H]+ | C11H6O3 | 131.049:1708 159.03799:514 187.036:6425 | 187.039 | Psoralen |
| 42.631 | 597.14709 | [M]+ | C26H29O16 | 303.04883:955 304.04895:263 | 597.14447 | Delphinidin-O-sambubioside |
| 43.373 | 465.09827 | [M]+ | C21H21O12 | 303.04901:11552 304.04901:1874 | 465.1022 | Delphinidin-galactoside |
| 44.874 | 433.11374 | [M+H]+ | C21H20O10 | 283.05899:8959 313.069:16247 314.073:3400 323.08899:1017 337.069:4448 351.08499:1828 , 367.08301:5487 379.077:4211 , 397.08701:10951 , 398.09299:2893 415.103:10873 416.103:2406 433.11401:7131 | 433.11362 | Isovitexin |
| 46.391 | 595.16791 | [M+H]+ | C27H30O15 | 287.05316:272 | 595.16534 | Lonicerin |
| 46.391 | 595.16791 | [M+H]+ | C27H30O15 | 287.05316:400 449.09262:51 450.11057:61 | 595.16699 | Nicotiflorin |
| 46.408 | 433.11374 | [M+H]+ | C21H20O10 | 287.05301:10229 288.056:1350 | 433.11292 | Kaempferol-O-rhamnoside |
| 47.198 | 433.11374 | [M+H]+ | C21H20O10 | 271.061:60604 272.065:12432 273.064:1820 | 433.11292 | Apigenin-O-glucoside |
| 47.206 | 447.08954 | [M+H]+ | C21H18O11 | 271.061:94594 271.30801:856 271.55399:332 272.065:21561 273.064:2868 | 447.08911 | Baicalin |
| 47.207 | 609.17688 | [M+H]+ | C28H32O15 | 129.05341:63 301.07169:4079 302.06854:752 303.07306:106 463.11966:1205 464.12344:392 465.1283:127 | 609.18207 | Diosmin |
| 47.212 | 301.07169 | [M+H]+ | C16H12O6 | 286.04599:2809 301.07199:25772 | 301.07071 | Scutellarein 4'-methyl ether |
| 47.219 | 303.04883 | [M+H]+ | C15H10O7 | 137.01801:3116 153.015:7702 229.047:14324 247.05701:6752 257.043:17322 285.035:11323 286.04599:2988 301.07199:12063 302.07501:2274 303.04901:1402930 303.48499:7412 | 303.05045 | Quercetin |
|  |  |  |  |  |  |  |
